# Controllable Fabrication of ZnO Nanorod Arrays on the Surface of Titanium Material and Their Antibacterial and Anti-Adhesion Properties

**DOI:** 10.3390/ma18071645

**Published:** 2025-04-03

**Authors:** Sifang Kong, Jialin Li, Ouyang Fan, Feng Lin, Jiayin Xie, Jing Lin

**Affiliations:** 1 School of Traffic & Environment, Shenzhen Institute of Information Technology, Shenzhen 518172, China; mengsiksf@163.com; 2 School of Chemistry and Chemical Engineering, Guangzhou University, Guangzhou 510006, China

**Keywords:** ZnO nanorod, titanium material, hexagonal wurtzite, antibacterial, anti-adhesion

## Abstract

The adhesion of deleterious bacteria on titanium substrates not only causes economic losses but also endangers human life and health. The study is expected to address the challenging issues of using ZnO as an antibacterial material, including low bactericidal efficiency without lighting, susceptibility to ZnO cluster formation, and easy adhesion of bacteria to its surface. It is proposed that the prepared ZnO nanorod arrays with a hexagonal wurtzite structure on the surface of titanium-based materials can address the issue of ZnO cluster formation. Remarkably, a mere 3.49 g cm^−2^ of decorated Ag/AgCl achieves over 99% sterilization efficiency without lighting. The incorporation of FAS (1H,1H,2H,2H-perfluorodecyltrimethoxysilane) molecules with low surface energy enables the prepared Ti@ZnO@Ag/AgCl@FAS to attain a Cassie–Baxter wetting state, thereby imparting exceptional bacterial anti-adhesion properties exceeding 99.50%. Furthermore, antibacterial and anti-adhesion models have been proposed to elucidate the underlying mechanisms. This innovative approach is anticipated to be adaptable for application across various material substrates, which opens up a new avenue for the application of the antibacterial and bacterial anti-adhesion properties on the surface of ZnO materials.

## 1. Introduction

Tackling the harm of deleterious bacteria has always been recognized as one of the most prominent issues in both human health and economic development [1]. Bacteria in water pipes can cause the deterioration of drinking water [2], while those on food equipment can cause food spoilage and foodborne illness [3,4,5]. In 2019, bacterial infections caused 1.27 million deaths [6], and without effective interventions, could claim the lives of a daunting 350 million people by the year 2050 [7]. Bacteria also form biofilms on surfaces, causing economic losses, such as microbial corrosion [8], which accounts for 20% of metal corrosion costs [9,10,11], with China losing RMB 400 billion annually. Thus, eliminating bacteria and preventing adhesion is vital for health and economic reasons.

Continuously developed antibacterial materials have demonstrated their effective bactericidal properties. These materials are primarily categorized into organic and inorganic types. Organic antibacterial materials encompass a wide range of compounds, including quaternary ammonium salts [12], phenols [13,14], guanides [15], organometallic compounds [16,17], natural compounds [18], chitosan and its derivatives [19], as well as antimicrobial peptides [20]. On the other hand, inorganic antibacterial materials predominantly consist of metal-based and carbon-based substances such as Ag [21,22], Cu [23], graphene [24], TiO_2_ [25], ZnO [26,27,28,29,30,31], CuO [32], ZrO_2_ [33], SnO_2_ [34], etc. Their widespread application has significantly contributed to safeguarding human health and mitigating economic losses. However, it is worth noting that organic antibacterial materials are susceptible to decomposition at elevated temperatures and may carry inherent toxicity concerns [35,36]. Inorganic metal nanomaterials offer advantages such as superior bactericidal efficacy and resistance to drug-resistant strains [37,38,39]. Zinc oxide (ZnO) is a notable inorganic metal antibacterial nanomaterial certified by the United States Food and Drug Administration (FDA) for safety in commercial applications [40,41]. Previous studies demonstrated that ZnO exhibited exceptional bacterial properties when exposed to light [42,43]. However, it was also revealed that ZnO demonstrated limited antibacterial efficacy in the absence of light [44]. Many researchers are currently exploring methods to enhance the light absorption capability of ZnO [45,46,47,48,49], such as forming heterojunction composites with metal oxides or doping with other ions. Moreover, the accumulation of dead bacteria on the surface despite eradication efforts may potentially contribute to biofilm formation [50]. Researchers have successfully engineered a superhydrophobic material surface endowed with bacterial anti-adhesion functionality [51,52,53]. The Cassie–Baxter wetting theory reveals that the surface contact angle of composite materials is influenced by two primary factors—the surface energy of the material and its surface roughness [54]—where the Cassie–Baxter State is a wetting state of a solid surface that occurs when a liquid droplet rests on a textured or patterned surface, and the liquid partially wets the surface by forming pockets of air between the droplet and the solid surface. Through the hydrothermal method, it is possible to create a specific morphology and structure of ZnO with a certain level of surface roughness. By adjusting the reaction conditions, one can control the growth height and diameter of ZnO, thereby accurately manipulating its morphology and size [55,56]. This allows for the creation of ZnO array microstructures on metal material surfaces, which, when combined with low-surface-energy modification, are expected to result in a material surface exhibiting low-adhesion characteristics in a Cassie–Baxter state [57,58,59].

**Figure 1:**
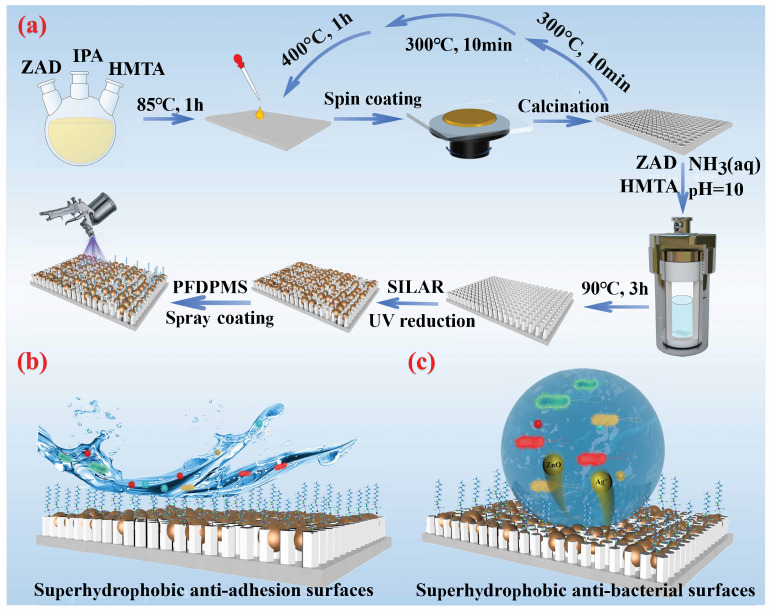
Synthesis process of antibacterial and bacterial anti-adhesive composites. (**a**) Fabrication process of Ti@ZnO@Ag/AgCl@FAS; (**b**) schematic diagram illustrating the anti-adhesion properties of Ti@ZnO@Ag/AgCl@FAS; (**c**) schematic diagram of the antibacterial activity of Ti@ZnO@Ag/AgCl@FAS.

Herein, we present a novel approach to the controllable fabrication of ZnO nanorod arrays exhibiting a hexagonal wurtzite structure on the surfaces of titanium-based materials. These structures are further enhanced through modification with Ag/AgCl and low-surface-energy FAS molecules, as illustrated in Figure 1a. The directional growth facilitated by the titanium-based substrate is anticipated to not only mitigate the formation of ZnO clusters but also promote effective sterilization in the absence of light due to the incorporation of Ag/AgCl. This innovative strategy addresses both issues: it resolves cluster formation while simultaneously ensuring efficient antibacterial action under dark conditions. Moreover, the introduction of low-surface-energy FAS molecules allows for the resultant Ti@ZnO@Ag/AgCl@FAS composite to achieve a Cassie–Baxter wetting state, thereby enhancing bacterial anti-adhesion properties. Ultimately, we propose comprehensive antibacterial and anti-adhesion models that elucidate the underlying mechanisms at play, as illustrated in Figure 1b,c. Importantly, this methodology is not confined solely to titanium-based substrates; rather, it holds promise for versatile applications across various material platforms. This breakthrough paves new pathways for advancing antibacterial strategies and mitigating bacterial adhesion on surfaces modified with ZnO materials.

## 2. Materials and Methods

### 2.1. Materials

The titanium substrate was procured from the local market, while 1H,1H,2H,2H-perfluorodecyltrimethoxysilane (FAS), hexamethyltetramine (HTMA), sodium chloride, and zinc acetate dihydrate (ZAD) were sourced from Macklin, Shanghai, China. Isopropyl alcohol, triethylamine, ammonia, anhydrous ethanol, nitric acid, silver nitrate, and hydrofluoric acid were obtained from Tianjin Damao Co., Ltd., Tianjin, China. The strains *E. coli* [ATCC(B) 25922] and *S. aureus* [CMCC(B) 26003] were acquired from the Shanghai Luwei Institute of Microbial Sciences, Shanghai, China. Additionally, phosphate-buffered brine (PBS), Mueller–Hinton AGAR (MHA), and Mueller–Hinton Broth (MHB) were purchased from Sigma-Aldrich, St. Louis, MO, USA.

### 2.2. Preparation of ZnO Seed Layer on Ti Substrate

To begin, the titanium plate is meticulously sliced into 2 × 2.5 cm thin segments, followed by immersion in 50 mL of polishing liquid (comprising hydrofluoric acid, nitric acid, and water at a ratio of 0.56 g:3.84 g:5 g (1 moL:2 moL:3 moL)) for a duration of two minutes. This chemical polishing process effectively eliminates any residual oil and oxide layers from the surface. Subsequently, the polished titanium plates undergo ultrasonic cleaning with anhydrous ethanol and deionized water for five minutes each before being placed in an oven at 80 °C for thorough drying. Upon completion of the drying process, zinc acetate dihydrate (0.224 g, 1.02 mmoL) and isopropyl alcohol (25 mL, 0.327 moL) are combined within a round-bottom flask. The temperature of the constant temperature magnetic water bath stirring pot is then adjusted to 85 °C for one hour while incorporating a rotor and initiating condensation. After allowing thirty minutes for reaction time, 555 μL (7 mmoL) of triethylamine is introduced to sustain stirring until a pale milk-white seed layer solution is achieved at the conclusion of the reaction. Following this step, the round-bottom flask is removed and allowed to cool prior to further use. The dried titanium plate proceeds to be positioned within a spinning coating machine, where it receives an application of the ZnO seed solution totaling 2 mL through spin coating adjustments set at rates of 600 rpm for ten seconds, followed by 800 rpm for five seconds, and finally culminating with 1200 rpm over twenty seconds. Subsequent calcination occurs within a Muffle furnace at 300 °C for ten minutes before repeating another cycle of spin coating under identical conditions post-cooling. Following this additional round of calcination at 300 °C for ten minutes, the titanium plate is once again removed for cooling, prior to repeating the spin-coating process and subjecting it to an hour-long calcination within the Muffle furnace at 450 °C. Ultimately, the calcined titanium plate is then allowed to cool before being sealed in a bag to shield it from light and stored in a dry environment until it is needed for use.

### 2.3. Preparation of Ti@ZnO Composite Material

Having measured 0.34 g (1.55 mmoL) of zinc acetate dihydrate and 0.21 g (1.5 mmoL) of hexamethylenetetramine, they were separately dissolved in 25 mL of distilled water before being combined in a polytetrafluoroethylene liner for thorough mixing. The pH level plays a crucial role in regulating the growth rate of nano-ZnO crystal faces. Upon adding 3 drops of ammonia water, the previously clear mixture transformed into a milky white substance resembling cotton, which was then transferred to the reaction vessel and subjected to an oven temperature of 90 °C for a duration of 3 h for the hydrothermal reaction process. Following this, the titanium plate was removed, meticulously washed with distilled water, dried, cooled down, and finally stored in a sealed bag for future use.

### 2.4. Preparation of Bacterial Anti-Adhesive Ti@ZnO@Ag/AgCl Composite Surface

In order to confer more efficient antibacterial properties on Ti@ZnO, Ag/AgCl was deposited onto its surface using the SILAR method. The specific steps are as follows: firstly, Ti@ZnO is soaked in a AgNO_3_ solution with concentrations of 0.17 g, 1 mmol L^−1^; 0.85 g, 5 mmol L^−1^; and 1.7 g, 10 mmol L^−1^ for 10 min, respectively, and then Ti@ZnO is transferred to deionized water for 2 min. Then, the surface of Ti@ZnO is transferred to a sodium chloride solution with corresponding concentrations of 0.12 g, 2 mmol L^−1^; 0.58 g, 10 mmol L^−1^; and 1.17 g, 20 mmol L^−1^ for 10 min, and then the surface of Ti@ZnO is transferred to deionized water for 2 min. This sequence was repeated thrice. The composite material loaded with AgCl was then subjected to irradiation under a 1000 W ultraviolet lamp for a duration of 10 min. As a semiconductor material, AgCl undergoes photoexcitation, facilitating the transition of electrons from the valence band to the conduction band. This process generates photogenerated electrons (e^−^) and holes (h^+^). The photogenerated electrons reduce the Ag^+^ ions in AgCl to metallic silver nanoparticles (Ag), while the holes oxidize Cl^−^ ions to Cl_2_, thereby leading to the partial reduction of AgCl to Ag [60]. The resulting samples were denoted as Ti@ZnO@Ag/AgCl(1), Ti@ZnO@Ag/AgCl(5), and Ti@ZnO@Ag/AgCl(10), where the numbers in parentheses corresponded to the concentration of the AgNO_3_ precursor solution used during deposition.

### 2.5. Preparation of Superhydrophobic-Type Antibacterial and Bacterial Anti-Adhesive Ti@ZnO@Ag/AgCl@FAS Composite Surface

The Ti@ZnO@Ag/AgCl obtained was subjected to modification with FAS in order to achieve the hydrophobic Ti@ZnO@Ag/AgCl@FAS. The specific steps were as follows: Initially, a mixed solution of FAS in ethanol with a mass fraction of 1 wt% was prepared. Subsequently, the pH was adjusted to 10 using a 25% ammonia solution. Following this, the mixed solution was sprayed onto the surface of Ti@ZnO@Ag/AgCl using a thermal spraying method. By controlling the spray volume, hydrophobic composites with grafted densities ranging from 1 to 5 mg cm^−2^ were obtained. The composites were then placed in an oven at 80 °C for 3 h to complete the process. The resulting samples were designated as Ti@ZnO@Ag/AgCl@FAS(1), Ti@ZnO@Ag/AgCl@FAS(2), Ti@ZnO@Ag/AgCl@FAS(3), Ti@ZnO@Ag/AgCl@FAS(4), and Ti@ZnO@Ag/AgCl@FAS(5), where the numbers in brackets corresponded to the graft density (mg/cm^2^, mass of grafted FAS on the surface per square centimeter of Ti@ZnO@Ag/AgCl) of FAS. The specific components of the as-prepared Ti@ZnO@Ag/AgCl@FAS composite materials are detailed in Table 1.

### 2.6. Characterization and Measurements

The surface morphology of the composites was observed using field-emission scanning electron microscopy (SEM, Sigma300, Zeiss, Jena, Germany). The crystal structure of the composites was characterized through X-ray diffraction (XRD, PW3040/60, PANalytical, Eindhoven, The Netherlands). Furthermore, the distribution of elements on the surface of the composite was examined using energy-dispersive spectrometry (EDS, Smartedx, Zeiss, Germany), while the surface chemical composition was analyzed via X-ray photoelectron spectrometry (XPS, K-Alpha+, Thermo Fisher Scientific, Waltham, MA, USA). In addition, the effect of surface roughness on the composite was studied using atomic force microscopy (AFM, Bruker Dimension ICON, Bruker, Santa Barbara, CA, USA). The loading of Ag/AgCl within the composite was determined with accuracy through inductively coupled plasma mass spectrometry (ICP-MS, ICPMS 7700, Agilent, Santa Clara, CA, USA). Moreover, to measure the water contact angle (WCA) and adhesion properties of the composite surface, sophisticated equipment, such as an optical contact angle meter (OCA40-Micro, Data physics, Filderstadt, Germany) and a highly sensitive MEMS balance system (DCAT11, Data Physics, Filderstadt, Germany) were employed.

### 2.7. Antibacterial Activity Assessment

In this study, *E. coli* was chosen as the representative of Gram-negative bacteria, while S. aureus was selected to represent Gram-positive bacteria for the purpose of examining the factors influencing the antibacterial properties of the composite. Initially, both the experimental sample and blank control (Ti) were cut into 1 × 1 cm squares and subsequently sterilized along with all experimental equipment. Following sterilization, these samples were placed into conical bottles on an aseptic operating table. Subsequently, 2 mL of a 10^7^ CFU mL^−1^ bacterial suspension and 48 mL of PBS buffer were added to each conical bottle before being transferred to an oscillating incubator set at 37 °C and 180 rpm for a duration of 6 h. Upon completion of incubation, a volume of 1 mL from each conical bottle was extracted and diluted by a factor of 100. Thereafter, 100 μL from each diluted bacterial solution was evenly spread onto AGAR medium plates, which were then cultured at a temperature of 37 °C for a period lasting up to twenty-four hours. At the conclusion of this cultivation period, colony counts within the medium were conducted followed by multiplication with dilution ratios in order to obtain the corresponding colony numbers. The antibacterial rate was calculated using Equation:

Antibacterial rate (%) = (CFU_control_ mL^−1^ − CFU_sample_ mL^−1^)/CFU_control_ mL^−1^

where CFU_control_ mL^−1^ denotes the mean number of bacteria per unit volume for the control sample across three repeated trials, and CFU_sample_ mL^−1^ denotes the mean number of bacteria per unit volume for the composite material over three replicates.

### 2.8. Bacterial Anti-Adhesive Assessment

The precise procedure of the anti-bacterial adhesion test is as follows: Initially, the experimental sample, the control (Ti), and the experimental equipment utilized are subjected to sterilization. Subsequently, the experimental samples and controls are placed into conical bottles on an aseptic operating table. Then, 10 mL of a bacterial suspension containing *E. coli* or *S. aureus* at a concentration of 10^7^ CFU mL^−1^ is added to the conical bottles. The conical bottles are then transferred to an oscillating incubator set at a controlled temperature of 37 °C and a rotating speed of 180 rpm for a duration of 2 h. Following incubation, the samples and blank controls are removed and placed in test tubes containing 5 mL of PBS. The test tubes are then positioned in an oscillating incubator at a controlled speed of 180 rpm for 30 min to desorb bacteria from the surface of the composite material. After oscillation, 100 μL of the bacterial solution is evenly spread onto AGAR medium from the test tube, followed by cultivation in an incubator at 37 °C for a period of 18 h. Upon completion of cultivation, colonies on the medium are counted. The bacterial anti-adhesion rate is determined by applying Equation for the calculation:

Bacterial anti-adhesion rate (%) = (CFU_control_ mL^−1^ − CFU_sample_ mL^−1^)/CFU_control_ mL^−1^

where the CFU_control_ mL^−1^ denotes the mean number of bacteria per unit volume for the control in three repeated experiments, and CFU_sample_ mL^−1^ denotes the mean number of bacteria per unit volume for the sample in three repeated experiments.

### 2.9. The Morphology of Bacteria Adhering on the Composite Surface

The adhesion morphologies of *E. coli* and *S. aureus* on the surface of the composite were observed using scanning electron microscopy (SEM). The experimental procedure involved conducting a bacterial anti-adhesion test on the composite material, followed by washing the bacteria-attached composite with 2 mL of PBS buffer for three consecutive cycles. Subsequently, the surface of the composite was treated with 30 μL of 2.5 wt% glutaraldehyde to fix the bacteria, which were then subjected to standing treatment for one hour. After fixation, the bacteria were washed twice with PBS buffer and once with distilled water. Gradient dehydration of the fixed bacteria was carried out using alcohol at concentrations of 25, 50, 75, 90, and 100 wt% for a duration of 15 min at each concentration. Finally, the treated samples were dried in a dryer before being observed via SEM.

### 2.10. Live/Dead Bacterial Viability Assay for Antibacterial and Bacterial Anti-Adhesive Assessment

The viability of bacteria in antimicrobial and anti-bacterial adhesion tests was assessed through visual observation using fluorescent staining. The antibacterial properties of the composite were judged by the ratio of survival and death of bacteria. The specific procedure involved taking 100 μL of the bacterial suspension onto a clean slide after the antibacterial or bacterial anti-adhesion test, followed by adding 25 μL of a mixture containing green SYTO9 fluorescent dye and red propyl iodide (PI) fluorescent dye to the bacterial suspension. After incubating in darkness for 15 min, a cover glass was applied, and the bacteria were examined using fluorescence microscopy (Olympus BX51, Olympus Co., Ltd., Tokyo, Japan) with green and red filters at laser emission bands of 440–480/515–540 nm and 540–560/630–660 nm, respectively.

## 3. Results and Discussion

### 3.1. Controllable Preparation and Growth Mechanism of Ti@ZnO Arrays

**Figure 2:**
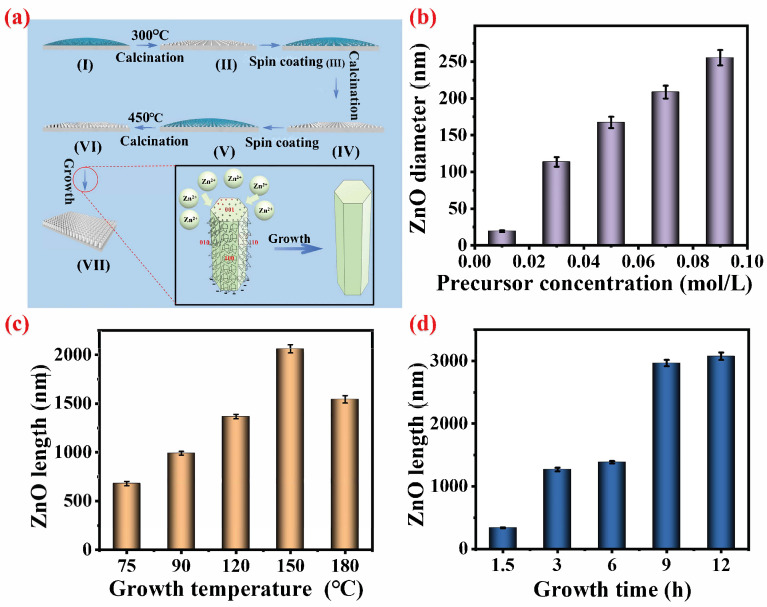
(**a**) Elucidation of the growth mechanism of ZnO exhibiting a hexagonal wurtzite structure; (**b**) effect of the precursor concentration on the diameter of ZnO with a hexagonal wurtzite structure; (**c**) effect of the hydrothermal growth temperature on the vertical growth height of ZnO displaying a hexagonal wurtzite structure; (**d**) effect of the hydrothermal growth time on the vertical growth height of ZnO with a hexagonal wurtzite structure.

The process diagram illustrating the controllable preparation of ZnO nanorod arrays with a hexagonal wurtzite structure is depicted in Figure 2a. Initially, during the seed nucleation stage of the seed layer, a nanometer ZnO seed sol is uniformly applied onto the surface of the titanium substrate through spin coating. Subsequent calcination at 300 °C (I) leads to heterogeneous nucleation between the titanium substrate and the non-polar (100) surface of ZnO, resulting in the distribution of spherical ZnO particles at specific deposition sites on the substrate’s surface. Following another round of spin coating with seed sol (II) and calcination (III), an increase in ZnO seed density on the titanium substrate surface is achieved (IV). The third application of spin-coated seed sol (V), coupled with a higher calcination temperature of 450 °C, facilitates particle–particle interactions among ZnO seeds and those deposited on the titanium substrate (Zn-OXO-acetate), effectively superseding particle–substrate (Ti) interactions, forming ZnO seed crystals (VI) [61,62]. This process promotes effective guidance for the crystal growth direction during subsequent hydrothermal crystal growth stages (VII). Subsequently, it enters into a growth stage characterized by ZnO crystals exhibiting a hexagonal wurtzite structure placed within a hydrothermal reactor containing precursor solution for hydrothermal crystal growth. The specific reactions involved in this hydrothermal growth process are as follows:

(1) $${\left({CH}_{2}\right)}_{6}N_{4}+6H_{2}O↔6HCHO+4{NH}_{3}$$

(2) $${NH}_{3}+H_{2}O↔{NH}_{4}^{+}+{OH}^{-}$$

(3) $${2OH}^{-}+{Zn}^{2+}↔{Zn(OH)}_{2}$$

(4) $${Zn(OH)}_{2}↔ZnO+H_{2}O$$

The underlying mechanism for the hydrothermal controlled preparation of ZnO with a hexagonal wurtzite structure can be elucidated as follows: The synthesis of ZnO necessitates the amalgamation of Zn^2+^ ions from the precursor and OH^−^ ions from the solution, resulting in the formation of Zn(OH)_2_ (3), which subsequently decomposes into ZnO (4) to facilitate continuous axial and radial growth on the surface of ZnO seed crystals. On one hand, the stable source of OH^−^ ions is derived from the added ammonia, which undergoes continuous consumption and hydrolysis into NH_3_ (1) by HMTA. This process ensures a sustained replenishment of OH^−^ (2) to maintain a specific pH environment. Furthermore, apart from serving as a source of OH^−^ ions in hydrothermal reactions, HMTA plays a crucial role as a non-polar chelating agent that selectively adsorbs onto the non-polar surfaces (010, 110, 100) of ZnO crystals. Consequently, this exposure only allows for epitaxial growth on the polar surface (001), thereby contributing significantly to the formation of hexagonal wurtzite structures in ZnO crystals [63].

The growth rate and length–diameter ratio of ZnO with a hexagonal wurtzite structure are primarily influenced by the concentration of the precursor zinc source, growth temperature, and growth time [41,55,56,64]. As depicted in Figure 2b and Figure S1a (ESI), a precursor concentration of 0.01 mol L^−1^ results in limited availability for ZnO growth, leading to the observation of only a seed layer (Figure S1a, ESI). Reaction (4) indicates that a lower Zn^2+^ concentration does not supply sufficient zinc source for the growth of ZnO nanoparticles, thus preventing the formation of ZnO nanorod arrays with a hexagonal wurtzite structure [65]. However, as the precursor concentration is gradually increased (0.03, 0.05, 0.07, 0.09 mol L^−1^), it enables the formation of ZnO nanorod arrays with a hexagonal wurtzite structure (Figure S1a, ESI). Specifically, at a concentration of 0.03 mol L^−1^, neat and sparse hexagonal wurtzite-structured ZnO nanorods are formed with gaps ranging from 40 to 400 nm. Moreover, when the concentration is increased to 0.05 mol L^−1^, an abundance of zinc sources promotes the growth of larger diameter hexagonal wurtzite ZnO nanorods with a gap size reaching approximately 200 microns. Furthermore, as the precursor concentration continues to increase progressively (from 0.03 to 0.09 mol L^−1^), there is a noticeable trend toward larger diameters and closer gap formations within ZnO nanorod arrays. Statistical analysis reveals that the corresponding diameters also significantly increase alongside this trend: specifically at concentrations of 112.23, 173.33, 208.33, and 265.56 nm, respectively (Figure 2b). Henceforth, it can be concluded that the concentration of the zinc source can effectively regulate the radial diameter and array gap of ZnO nanorods with a hexagonal wurtzite structure [66].

The influence of the growth temperature on the development of ZnO nanorod arrays with a hexagonal wurtzite structure was further explored. As depicted in Figure S1b (ESI), it is evident that as the growth temperature (75, 90, 120, 150, and 180 °C) increases, the growth of ZnO nanorods is enhanced. The corresponding growth heights of the resulting ZnO nanorod arrays are 679.69, 990.42, 1366.17, 2052.17, and 1573.86 nm, respectively, which show a trend of first increasing and then decreasing. This suggests that the influence of the growth temperature on the polar surface of ZnO crystals (0010) outweighs that on other non-polar surfaces. This phenomenon may be attributed to the acceleration effect of a temperature increase on Zn^2+^ ion movement and the ZnO formation rate being more pronounced for non-polar surfaces due to their coverage by the chelated HMTA, which mitigates this effect to some extent, resulting in a faster growth rate in the axial direction than in the radial direction [67]. As depicted in Figure S1c (ESI), it is evident that at elevated growth temperatures of 150 and 180 °C, the hexagonal wurtzite structure exhibits a pronounced sharpness when the temperature exceeds 150 °C. This phenomenon can be attributed to the interplay between the axial and radial growth rates of ZnO crystals, which are delicately balanced by temperature [68]. Upon reaching 180 °C, there is a decrease in growth height, possibly due to the reversible nature of Reactions (1–4), leading to an equilibrium shift at excessively high temperatures. The reaction temperature surpasses the critical threshold for ZnO growth, thereby impeding its favorable development [63]. Consequently, it is apparent that precise control over the growth temperature can effectively govern both the vertical extent and topographical morphology of ZnO structures.

The effect of the growth time on the development of ZnO nanorod arrays with a hexagonal wurtzite structure was further investigated. The duration of growth plays a pivotal role in regulating the vertical growth dimension of ZnO. As depicted in Figure S1d (ESI), an escalation in the growth time (1.5 h, 3 h, 6 h, 9 h, and 12 h) corresponds to an increase in the height of ZnO nanorods. Specifically, the resultant heights for the hexagonal wurtzite ZnO nanorod arrays were observed to be 338.80, 990.42, 1399.70, 2916.98, and 3089.89 nm, respectively. Figure S1d (ESI) illustrates the growth of ZnO nanorod arrays at the nucleation site of ZnO seed crystals, with no formation of new nanostructures by ZnO. The incomplete and uneven growth state is evident in the early stage (1.5 h), characterized by a non-growing seed layer at the bottom. As time progresses, the array of hexagonal wurtzite nanorods grows neatly and sparsely within 3–12 h. According to the growth theory, when the zinc source is saturated, Reactions (3) and (4) tend toward ZnO deposition on pre-existing ZnO seed crystals, resulting in a continued increase in the ZnO growth height over time [69]. However, beyond 9 h of growth time, there is no significant increase in the growth height. The reason may be that the chelation and continuous hydrolysis of the non-polar surface of HMTA lead to the depletion of hydrolyzable HMTA, and the generated OH^−^ may be consumed, thereby limiting further growth height increases [63].

In summary, the chelation of HMTA on the non-polar surface of ZnO seed crystals plays a pivotal role in the formation of the hexagonal wurtzite structure. Furthermore, it is worth noting that the concentration of the precursor zinc source serves as the primary determinant in regulating both the radial diameter and array gap of hexagonal ZnO. Additionally, it is imperative to acknowledge that the growth temperature exerts an influence over not only the growth height but also the top morphology of ZnO, thereby influencing its surface roughness. Lastly, it should be emphasized that the growth time stands out as a key factor in controlling the growth height of ZnO. With an extension in the growth duration, one can observe that the development of the hexagonal wurtzite structure ZnO nanorod arrays becomes increasingly orderly yet sparse; concurrently, there is a gradual elevation in both the height of this array and its surface roughness. These three factors intricately interact to shape the surface microstructure, consequently impacting its hydrophobic properties.

### 3.2. Regulation of Surface Wettability of Ti@ZnO@FAS

It is important to investigate the surface wettability of ZnO nanorod arrays, as it profoundly impacts the interaction between bacterial droplets and the surface, thereby influencing the anti-bacterial adhesion properties of the surface. In the superhydrophobic Cassie–Baxter wetting state, a larger water contact angle signifies a reduced solid–liquid contact area for droplets on the surface, consequently lowering the likelihood of bacterial contact and adherence. Furthermore, due to its strong repellent effect on water, a superhydrophobic surface forms an air layer when immersed in water, effectively blocking bacteria and preventing their adhesion. Achieving a superhydrophobic Ti@ZnO@FAS surface in Cassie–Baxter state with high-contact-angle characteristics necessitates a synergistic interplay between surface microstructure and low surface free energy. As previously mentioned, the directional growth of ZnO yields a hexagonal wurtzite microstructure, while grafting the FAS structure onto the surface modifies its influencing conditions for achieving superhydrophobicity in the Cassie–Baxter state. As previously mentioned, the influence conditions on the superhydrophobic surface in the Cassie–Baxter state were further investigated through the controlled growth of ZnO with a hexagonal wurtzite microstructure and the graft modification of FAS on its surface.

**Figure 3:**
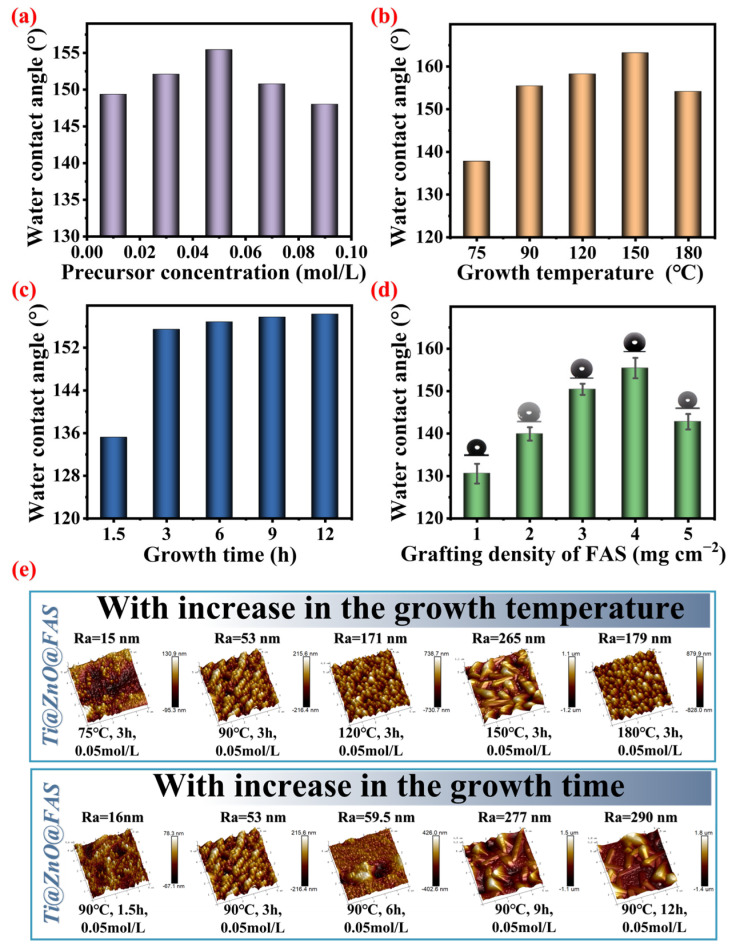
(**a**) Effect of the precursor concentration on the water contact angle of the Ti@ZnO@FAS surface; (**b**) effect of the growth temperature on the water contact angle of the Ti@ZnO@FAS surface; (**c**) effect of the growth time on the water contact angle of the Ti@ZnO@FAS surface; (**d**) effect of the grafting density of FAS on the water contact angle of the Ti@ZnO@FAS surface; (**e**) effect of the growth temperature and growth time on the surface roughness of Ti@ZnO.

Given that the growth conditions have a significant impact on the surface microstructure and, consequently, the surface wetting state, an investigation was conducted to explore the influence of the precursor concentration, growth temperature, and growth time on surface wettability. As depicted in Figure 3a, it was observed that as the precursor concentration (0.01, 0.03, 0.05, 0.07, and 0.09 mol L^−1^) increased continuously with the same amount of FAS being grafted onto the surface, the water contact angle initially increased and then decreased (149.4°, 152.1°, 155.4°, 150.8°, and 148.0°, respectively), which is obtained from the real water contact angle images (Figure S2, ESI). This phenomenon can be attributed to the fact that when the precursor concentration is at the 0.01 mol L^−1^ level or lower, the ZnO generated remains relatively short and retains its seed layer, resulting in ZnO nanorod arrays without a hexagonal wurtzite structure (Figure S1a, ESI), which has limited air capture ability and fails to form the superhydrophobic wetting state on its surface; However, ZnO nanorod arrays with a hexagonal wurtzite structure exhibit effective control over their surface microstructure (roughness and porosity) based on the precursor concentration, thereby regulating Ti@ZnO@FAS’s surface wettability. When the concentration of the zinc source is 0.05 mol L^−1^, the surface of Ti@ZnO@FAS demonstrates its highest level of hydrophobicity. Subsequently, an exploration was conducted to investigate the impact of the growth temperature on surface wettability, as depicted in Figure 3b. With an increase in the growth temperature (75, 90, 120, 150, and 180 °C), a consistent amount of FAS was grafted onto the surface. The water contact angle on the ZnO nanorods’ surface initially increased and then decreased (137.8°, 155.4°, 158.2°, 163.2°, and finally settling at 154.1°, which is obtained from the real water contact angle images (Figure S2, ESI)) due to the effective control exerted by the growth temperature over both the height and top morphology of ZnO nanorod arrays. Furthermore, as illustrated in Figure 3e, it can be observed that with an increase in the hydrothermal reaction temperature (75, 90, 120, 150, and 180 °C), the *R_a_* value on Ti@ZnO@FAS surface also exhibited a similar trend: first increasing and then decreasing (15, 53, 171, 265, and 179 nm, respectively). Notably, when the growth temperature reached a level of 150 °C, the resulting surface roughness was at its peak, which consequently led to maximal hydrophobicity for Ti@ZnO@FAS when grafted with equivalent amounts of FAS. Furthermore, as the hydrothermal growth time increases (1.5, 3, 6, 9, and 12 h) (Figure 3c), the water contact angle of the Ti@ZnO@FAS surface initially rises and then reaches a state of equilibrium (corresponding contact angles: 135.2°, 155.4°, 156.8°, 157.7°, and 158.3°, which is obtained from the real water contact angle images (Figure S2, ESI)). This phenomenon can be attributed to the continuous increase in both the height and diameter of the hexagonal wurtzite ZnO nanoarray generated with a prolonged growth time (1.5, 3, 6, 9, and 12 h). Consequently, the Ra values on Ti@ZnO@FAS also exhibit an initial upward trend (the *R_a_* values were 16, 53, 59.5, 277, and 290 nm, respectively), indicating a tendency toward increased hydrophobicity on the Ti@ZnO@FAS surface at equivalent FAS grafting levels. Finally, the impact of varying amounts of FAS with low surface energy on composite surface wettability was investigated. As depicted in Figure 3d, when the FAS grafting densities were set at 1, 2, 3, 4, and 5 mg cm^−2^, respectively, the contact angles for Ti@ZnO@FAS_(1)_, Ti@ZnO@FAS_(2)_, Ti@ZnO@FAS_(3)_, Ti@ZnO@FAS_(4)_, and Ti@ZnO@FAS_(5)_ were 130.6°, 139.9°, 150.4°, 155.4°, and 142.8°, respectively. It is evident that as the FAS grafting density increases from 1 to 4 mg cm^−2^, the composite surface contact angle gradually rises before declining once again as the grafting density surpasses 5 mg cm^−2^. This occurrence may be attributed to excessive grafting density leading to oil film formation, which partially covers the ZnO microstructure, thereby impacting the surface contact angle.

Through an investigation into the surface microstructure controlled by growth conditions and the modification of low surface energy, it has been demonstrated that the surface wettability of Ti@ZnO@FAS can be effectively regulated. This regulation results in the attainment of a superhydrophobic Cassie–Baxter wettability state for the surface of Ti@ZnO@FAS. The optimal growth conditions for Ti@ZnO have been determined as follows: a precursor concentration of 0.03 mol L^−1^, a growth temperature of 90 °C, a growth time of 3 h, and a low-surface-energy FAS modification amount of 4 mg cm^−2^ on the surface.

### 3.3. Characterization of Optimized Ti@ZnO@Ag/AgCl@FAS

**Figure 4:**
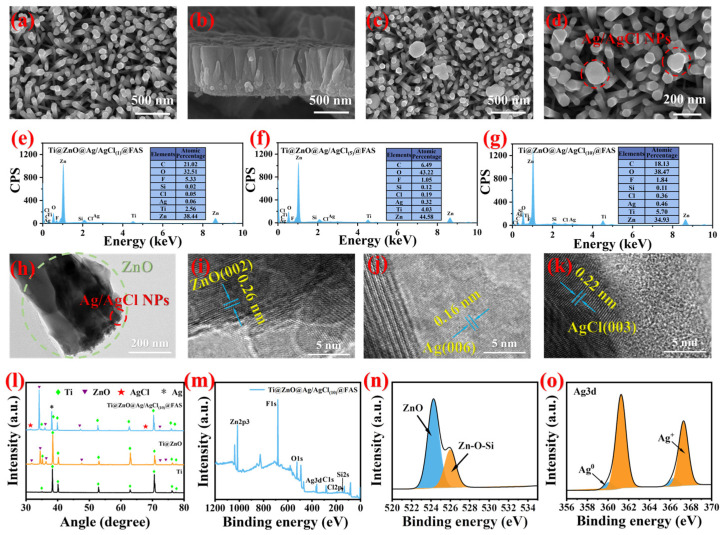
(**a**) Top-view SEM image of Ti@ZnO. (**b**) Cross-sectional SEM image of Ti@ZnO. (**c**) Top-view SEM image of Ti@ZnO@Ag/AgCl_(10)_. (**d**) Local high-magnification image of (**c**). (**e**) SEM-EDS mapping image of Ti@ZnO@Ag/AgCl_(1)_@FAS. (**f**) SEM-EDS mapping image of Ti@ZnO@Ag/AgCl_(5)_@FAS. (**g**) SEM-EDS mapping image of Ti@ZnO@Ag/AgCl_(10)_@FAS. (**h**) Transmission electron microscopy image of Ti@ZnO@Ag/AgCl_(10)_@FAS. (**i**) Lattice fringes of ZnO. (**j**) Lattice fringes of Ag. (**k**) Lattice fringes of AgCl. (**l**) XRD patterns of Ti, Ti@ZnO, and Ti@ZnO@Ag/AgCl_(10)_@FAS. (**m**) XPS survey spectrum of Ti@ZnO@Ag/AgCl_(10)_@FAS. (**n**) High-resolution XPS spectrum of O1s(Blue peak: Zn-O bond; Orange peak: Zn-O-Si bond). (**o**) High-resolution XPS spectrum of Ag3d(Blue peak:: Ag^0^; Orange peak: Ag^+^).

In order to enhance its antibacterial activity and bestow antibacterial adhesion properties upon Ti@ZnO, composite nanoparticles of antibacterial Ag/AgCl and modified low-surface-energy FAS were further deposited onto the ZnO nanorod arrays’ surface to achieve enhanced antibacterial adhesion. As depicted in Figure 4a, prior to the modification with Ag/AgCl, ZnO nanorod arrays with a hexagonal wurtzite structure having a side length of approximately 107 nm and a growth height of about 681.1 nm were grown on the surface of Ti@ZnO (Figure 4b). Following the modification with Ag/AgCl, as shown in Figure 4c, composite nanoparticles of Ag/AgCl were loaded onto the surface of Ti@ZnO@Ag/AgCl, with an approximate distribution size ranging between 20 and 250 nm (Figure 4d). Previous research has indicated that varying loads of Ag/AgCl can impact its antibacterial activity. Consequently, samples of Ti@ZnO@Ag/AgCl_(1)_@FAS, Ti@ZnO@Ag/AgCl_(5)_@FAS, and Ti@ZnO@Ag/AgCl_(10)_@FAS with different loads of Ag/AgCl were prepared by regulating the concentration of the precursor solution for silver nitrate (SEM-EDS mapping results displayed in Figure 4e), demonstrating uniform distribution on the surface area for elements such as Ag, Cl, F, Si, O, C, Ti, and Zn. By adjusting the solution concentration from 1 to 5 mmol L^−1^, and then to 10 mmol L^−1^, we could increase the silver content on the surface load, specifically resulting in element contents for silver at levels measuring at 0.06%, 0.32%, and 0.46%, respectively (SEM-EDS maps of specimens in Figure S3 (ESI)). Furthermore, HRTEM (high-resolution transmission electron microscopy) revealed the presence of Ag/AgCl composite particles loaded at the terminus of ZnO nanorods (Figure 4h). The lattice fringe spacing of ZnO was subsequently measured at 0.26 nm (Figure 4i), and upon comparison with the PDF card (JCPDS NO. 99-0111), it was unequivocally identified as the ZnO (002) crystal surface. Further scrutiny of the lattice structure of Ag/AgCl composite particles unveiled two distinct types of lattice fringes with spacings of 0.16 nm (Figure 4j) and 0.22 nm (Figure 4k). By cross-referencing with the PDF cards (JCPDS NO. 87-0598 and JCPDS NO. 22-1326), it can be conclusively affirmed that they correspond to the Ag (006) crystal face and AgCl (003), thus confirming the presence of Ag/AgCl composite nanoparticles. The crystal structure is further examined through XRD analysis. For the Ti plate, the characteristic peaks at the 35.093°, 38.412°, 40.170°, 53.004°, 62.949°, 70.660°, 74.157°, and 76.218° positions are identified as the (100), (002), and (002) Ti crystal faces corresponding to the PDF card (JCPDS NO. 44-1294). As for Ti@ZnO, the distinctive peaks at 31.766°, 34.4°, 36.3°, 47.5°, 72.6°, and 77.0° are determined to be ZnO’s (100), (002), (101), (102), (004), and (202) crystal faces in comparison with the PDF card (JCPDS NO.99-0111), indicating the formation of the hexagonal wurtzite structure ZnO [70]. In comparison to Ti@ZnO@Ag/AgCl, the characteristic peaks observed at 31.6° and 66.2° can be attributed to the (003) and (203) crystal faces of AgCl, as determined via a comparison with the PDF card (JCPDS NO. 22-1326). This further confirms the successful loading of Ag/AgCl composite particles onto the surface of Ti@ZnO. The surfaces of Ti@ZnO@Ag/AgCl were treated with low-surface-energy FAS, and an analysis of the surface elements and chemical bonds was conducted using XPS. The comprehensive XPS spectrum in Figure 4m reveals the presence of all expected elements (Ag, Cl, F, Si, O, C, Ti, and Zn) on the surface of Ti@ZnO@Ag/AgCl_(10)_@FAS. Through peak fitting analysis of the O and Ag elements and their bonding configurations with other elements, the high-resolution XPS map of O1s (Figure 4n) demonstrates that the distinct peaks at 524.3 and 525.9 eV correspond to Zn-O and Zn-O-Si bonds, indicating the attachment of FAS molecules to ZnO. The high-resolution XPS graph of Ag3d (Figure 4o) reveals the distinct peaks at 361.2 and 367.8 eV, corresponding to the energy levels of Ag^+^ in AgCl, specifically the Ag 3d5/2 and Ag 3d2/3 levels. Additionally, the peaks at 360.1 and 368.1 eV are indicative of the presence of the Ag element, showcasing its Ag 3d5/2 and Ag 3d3/2 levels, again proving the existence of Ag/AgCl.

### 3.4. Effects of Different Ag/AgCl Loading Amounts and Surface Wettability on Antibacterial Efficacy

**Figure 5:**
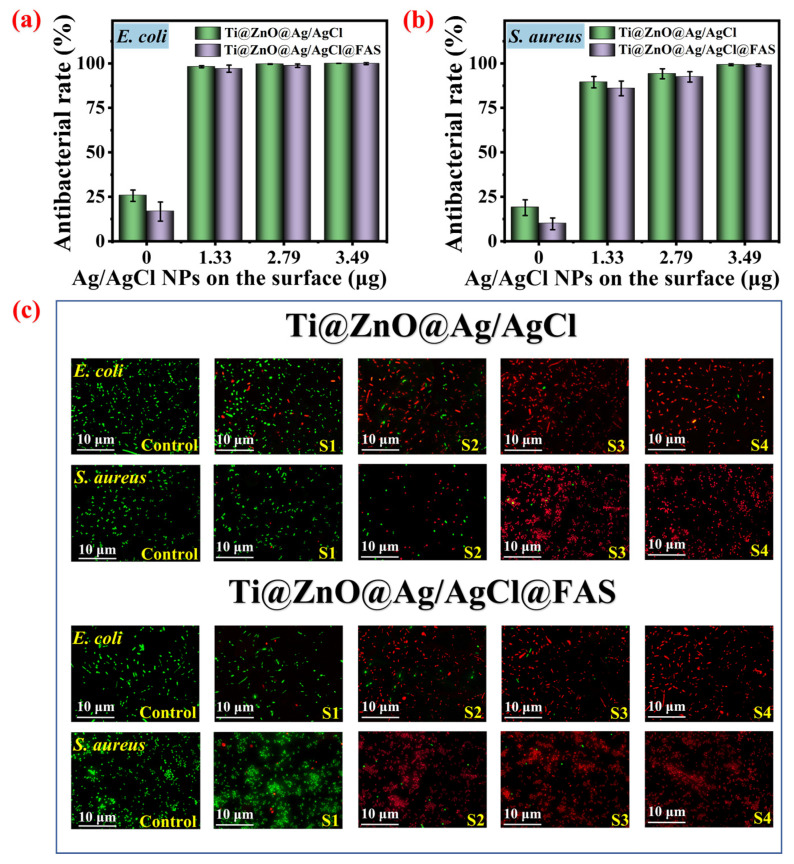
(**a**) Antibacterial rates of Ti@ZnO@Ag/AgCl and Ti@ZnO@Ag/AgCl@FAS samples against *E. coli*. (**b**) Antibacterial rates of Ti@ZnO@Ag/AgCl and Ti@ZnO@Ag/AgCl@FAS samples against *S. aureus*. (**c**) Fluorescence microscopic comparison of bacterial suspension after antimicrobial testing of the control and Ti@ZnO@Ag/AgCl samples (control represents Ti, S1 represents Ti@ZnO, S2 represents Ti@ZnO@Ag/AgCl_(1)_, S3 represents Ti@ZnO@Ag/AgCl_(5)_, and S4 represents Ti@ZnO@Ag/AgCl_(10)_), and fluorescence microscopic comparison of bacterial suspension after antimicrobial testing of the control and Ti@ZnO@Ag/AgCl@FAS samples (control represents Ti, S1 represents Ti@ZnO@FAS, S2 represents Ti@ZnO@Ag/AgCl_(1)_@FAS, S3 represents Ti@ZnO@Ag/AgCl_(5)_@FAS, and S4 represents Ti@ZnO@Ag/AgCl_(10)_@FAS).

Previous studies have demonstrated that the antibacterial efficacy of ZnO is significantly enhanced in the presence of light, attributed to its wide-band gap semiconductor oxide properties. Conversely, ZnO exhibit a narrower wide-band gap, limiting its response to only ultraviolet radiation [57]. This necessitates specific ultraviolet irradiation conditions to stimulate ROS for efficient sterilization, resulting in low light utilization and antibacterial efficiency under visible light conditions [59]. Here, the enhancement effect of different amounts of Ag/AgCl loaded onto the surface of Ti@ZnO arrays on ZnO sterilization in the absence of light is investigated. The loading capacity of Ag/AgCl was assessed using ICP-MS. The loading capacities of Ti@ZnO@Ag/AgCl_(1)_, Ti@ZnO@Ag/AgCl_(5)_, and Ti@ZnO@Ag/AgCl_(10)_ were determined to be 1.33, 2.79, and 3.49 μg cm^−2^, respectively. The oscillating culture method was utilized to compare the antibacterial properties under no-light conditions. The results of the plate colony counting method are presented in Figure S4 (ESI). As depicted in Figure 5, Ti@ZnO loaded with varying Ag/AgCl contents (Ti@ZnO@Ag/AgCl_(1)_, Ti@ZnO@Ag/AgCl_(5)_, and Ti@ZnO@Ag/AgCl_(10)_) exhibited antibacterial rates against *E. coli* of 25.64%, 98.17%, 99.63%, and 99.99%, respectively (Figure 5a). The corresponding antibacterial rates against *S. aureus* were 18.9%, 89.45%, 94.18%, and 99.26% (Figure 5b). It is evident that the antibacterial rate of Ti@ZnO@Ag/AgCl loaded with Ag/AgCl was significantly higher than that of Ti@ZnO without AgCl, indicating an increase in antibacterial properties with the augmentation of the Ag/AgCl load on the surface. After hydrophobic modification, the antibacterial rates of Ti@ZnO@FAS, Ti@ZnO@Ag/AgCl_(1)_@FAS, Ti@ZnO@Ag/AgCl_(5)_@FAS, and Ti@ZnO@Ag/AgCl_(10)_@FAS against *E. coli* were 16.69%, 97.05%, 98.71%, and 99.86%, respectively. The corresponding antibacterial rates against *S. aureus* were 9.82%, 85.95%, 92.46%, and 99.03%, respectively. Similarly, the augmentation of the Ag/AgCl load led to a substantial enhancement in the antibacterial properties of the Ti@ZnO@Ag/AgCl@FAS surface. In comparison to Ti@ZnO@Ag/AgCl prior to FAS spraying, the antibacterial efficacy of Ti@ZnO@Ag/AgCl@FAS exhibited a slight decrease, possibly attributed to the fact that the superhydrophobic wettability had a slight effect on the release of antibacterial ions Zn^2+^, ZnO, AgCl, and Ag^+^), thus affecting its overall antibacterial performance [53]. It is noteworthy that the antibacterial efficacy of the same sample against *E. coli* is marginally higher than that against *S. aureus*. This can be attributed to the difference in peptidoglycan thickness on the cell walls of Gram-negative and Gram-positive bacteria. The peptidoglycan thickness on the cell wall of Gram-negative bacteria ranges from 1 to 2 nm, whereas it measures 15–80 nm on the cell walls of Gram-positive bacteria. As a result, *E. coli*, having thinner cell walls, is more susceptible to eradication [71]. Furthermore, the bacterial solution post-antibacterial test underwent a fluorescence staining experiment to observe the status of dead and live bacteria using a fluorescence microscope (where dead bacteria appeared red, and live bacteria appeared green). As depicted in Figure 5c, the findings also proved that as the loading capacity of Ag/AgCl increased, there was a gradual rise in the proportion of dead bacteria following antibacterial tests on Ti@ZnO@Ag/AgCl and Ti@ZnO@Ag/AgCl@FAS. The results of the antibacterial performance were consistent with those of the antibacterial rate. All in all, the loading quantities of Ag/AgCl and the surface wettability are pivotal factors that significantly influence antibacterial efficacy.

**Figure 6:**
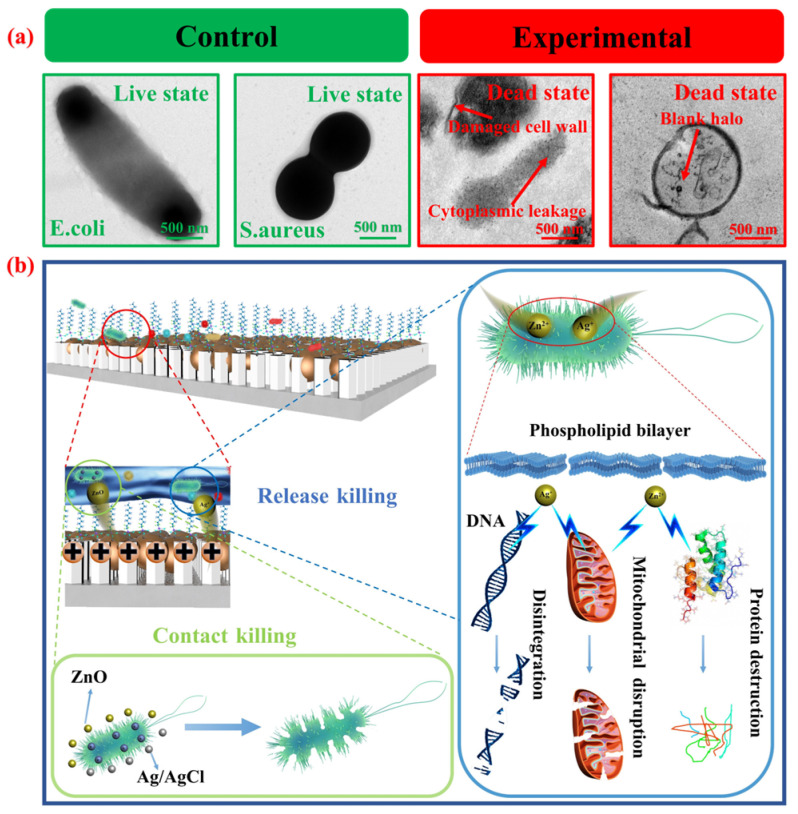
(**a**) Transmission electron microscopy images of *E. coli* and *S. aureus* in the control, as well as those of *E. coli* and *S. aureus* after undergoing antibacterial testing; (**b**) schematic diagram illustrating the bactericidal mechanism of Ti@ZnO@Ag/AgCl@FAS.

To investigate the interaction between Ti@ZnO@Ag/AgCl@FAS and bacteria, as well as the underlying bactericidal mechanism, the microscopic morphology of the bacteria was observed using biological transmission electron microscopy (Figure 6a). The control group consisted of non-sterilized *E. coli* and *S. aureus*, which exhibited intact cell walls and membranes. In contrast, after adding the Ti@ZnO@Ag/AgCl@FAS sample to the bacterial suspension for oscillating culture for 6 h, it was observed that the cell wall of the experimental group appeared deformed and broken, with a destroyed cell membrane and leaked cytoplasm. The overall shape of the *E. coli* cells was dismembered, while *S. aureus* cells were hollowed out. This severe destruction of their own structure led to the death of the bacteria, confirming the high bactericidal performance of Ti@ZnO@Ag/AgCl@FAS. In order to elucidate the underlying bactericidal mechanism, a bactericidal mechanism model Ti@ZnO@Ag/AgCl@FAS was proposed to further explicate the specific bactericidal process, as depicted in Figure 6b. The primary bactericidal mechanisms of Ti@ZnO@Ag/AgCl@FAS were contact and release bactericidal. The contact sterilization mechanism is attributed to the presence of numerous ZnO and Ag/AgCl composites with diameters of tens of nanometers on the surface of Ti@ZnO@Ag/AgCl@FAS. The antibacterial ZnO and Ag/AgCl may induce physical damage to the bacterial cell membrane upon collision with bacteria due to nano-effects during oscillatory culture, resulting in bacterial death [72,73]. Furthermore, ZnO and Ag/AgCl in the solution system will release Zn^2+^ and Ag^+^ ions, which, on one hand, interact with the negative charge on the surface of the bacterial cell membrane, thereby disrupting charge balance and causing severe cell deformation leading to bacterial lysis [74]; on the other hand, Zn^2+^ and Ag^+^ ions penetrate into the cell membrane, disrupting DNA transcription and translation in the cytoplasm, impairing mitochondrial respiratory chain enzymes, inhibiting protein synthesis, and ultimately disrupting cytoplasm metabolism within the cell membrane, resulting in bacterial death [75,76].

### 3.5. The Effect of the FAS Grafting Amount on the Bacterial Anti-Adhesion Rate of Ti@ZnO@Ag/AgCl@FAS

After the conventional antibacterial materials have eradicated bacteria, it is often observed that the dead bacterial cells remain adhered to the material’s surface. This phenomenon may obstruct the release channel of antibacterial active substances, impeding their contact and subsequent release. Consequently, this impediment weakens the sustainable and enduring antibacterial efficacy of the material. Furthermore, these dead bacterial cells can serve as new adhesion sites, thereby heightening the risk of bacterial proliferation and biofilm formation. Hence, an ideal antibacterial material should not only possess bactericidal properties but also exhibit characteristics that prevent dead bacteria from adhering to its surface. The resistance of bacterial adhesion is jointly determined by the low surface energy and microstructure of the material surface. Therefore, different amounts of FAS grafted on the surface of the microstructure were modified, and the effects on the antimicrobial adhesion rate of Ti@ZnO@Ag/AgCl@FAS were investigated. The oscillating culture method was employed to conduct a comparative assessment of the anti-bacterial adhesion rate, while the plate colony counting method was utilized to retroactively calculate its anti-adhesion rate (as illustrated in Figure S5 (ESI)).

**Figure 7:**
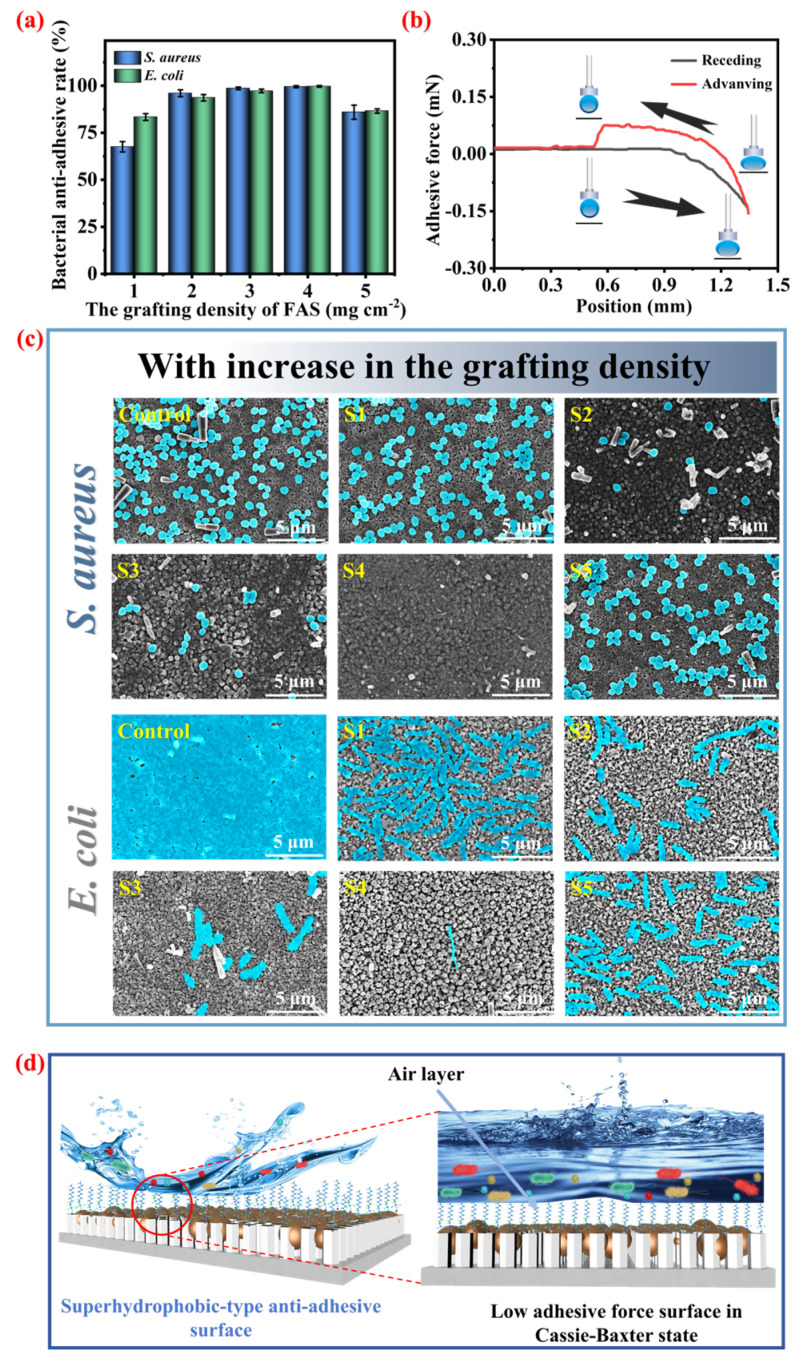
(**a**) Effect of FAS grafting amount on the bacterial anti-adhesion rate of Ti@ZnO@Ag/AgCl@FAS; (**b**) adhesion force test conducted on the surface of Ti@ZnO@Ag/AgCl@FAS; (**c**) SEM images of the distribution of adhered bacteria on the surface of Ti@ZnO@Ag/AgCl@FAS grafted with different FAS amounts after bacterial anti-adhesion test; (**d**) schematic illustration of the bacterial anti-adhesion mechanism of hydrophobic-type Ti@ZnO@Ag/AgCl@FAS.

As shown in Figure 7a, it is evident that varying the graft densities of FAS at 1, 2, 3, 4, and 5 mg cm^−2^ resulted in different antimicrobial adhesion rates to *E. coli* for Ti@ZnO@Ag/AgCl@FAS_(1)_, Ti@ZnO@Ag/AgCl@FAS_(2)_, Ti@ZnO@Ag/AgCl@FAS_(3)_, Ti@ZnO@Ag/AgCl@FAS_(4)_, and Ti@ZnO@Ag/AgCl@FAS_(5)_. The observed rates were found to be 67.58%, 96.02%, 98.64%, 99.50%, and 85.94%, respectively. Similarly, the antimicrobial adhesion rates to *S. aureus* were recorded as being at levels of 83.36%, 93.62%, 97.26%, 99.70%, and 86.59%, respectively. The distribution of bacteria adhered to the surface of Ti@ZnO@Ag/AgCl@FAS can be visually observed through SEM (Figure 7c). A substantial quantity of *E. coli* and *S. aureus* was found attached to the unmodified sample surface. As the graft density of FAS increased from 1 to 4 mg cm^−2^, there was a gradual reduction in the number of bacteria adhering to the Ti@ZnO@Ag/AgCl@FAS surface, followed by an increase when the graft concentration reached 5 mg cm^−2^. This phenomenon may be attributed to excessive FAS grafting. The presence of excessive FAS led to a blockage within the gaps of the ZnO nanorod arrays, thereby compromising its air-capturing capability and ultimately resulting in heightened bacterial adhesion. Therefore, after conducting an analysis of Ti@ZnO@Ag/AgCl@FAS_(1)_, Ti@ZnO@Ag/AgCl@FAS_(2)_, Ti@ZnO@Ag/AgCl@FAS_(3)_, Ti@ZnO@Ag/AgCl@FAS_(4)_, and Ti@ZnO@Ag/AgCl@FAS_(5)_ using a surface adhesion test (Figure S6, ESI), it was observed that when 4 mg cm^−2^ of FAS was grafted, the adhesive force of Ti@ZnO@Ag/AgCl@FAS_(4)_ was only 0.075 mN (Figure 7b). However, with the grafting of 5 mg cm^−2^ FAS, the adhesive force of Ti@ZnO@Ag/AgCl@FAS_(5)_ increased to 0.101 mN (Figure S6d, ESI). This observation is also consistent with the reduction in the surface contact angle described earlier (Figure 3d), transitioning from superhydrophobic to hydrophobic properties. Furthermore, the bacterial anti-adhesion mechanism of Ti@ZnO@Ag/AgCl@FAS is further elucidated through a model (Figure 7d). Upon contact with droplets or sewage-containing bacteria, the surface of the material effectively repels bacterial adhesion. Simultaneously, as water droplets roll off this superhydrophobic material, they carry away any attached pollutants (bacteria), thereby maintaining the cleanliness of the material’s surface. This remarkable performance can be attributed to the superhydrophobic Cassie–Baxter wetting state exhibited by Ti@ZnO@Ag/AgCl@FAS, which imparts exceptional hydrophobicity and minimal adhesion. The strong repellent effect exerted on water droplets by this superhydrophobic material is evident in the local magnified image: when water containing bacteria comes into contact with the material’s surface, a stable air layer forms that effectively prevents direct contact between the bacteria and the surface, resulting in outstanding anti-bacterial adhesion performance.

## 4. Conclusions

In conclusion, a controllable preparation of ZnO nanorod arrays with a hexagonal wurtzite structure on the surface of a titanium substrate has been developed to achieve efficient antibacterial and antibacterial adhesion in the absence of lighting. By manipulating the precursor concentration, growth time, and temperature, the controllable preparation of ZnO nanorod arrays can be achieved, thereby addressing issues such as clustering and creating specific microstructures on titanium-based materials. The chelation of HMTA on the non-polar surface of ZnO seeds plays a crucial role in facilitating the formation of the hexagonal wurtzite structure. The precursor concentration primarily regulates the radial diameter and array gap, while the growth temperature affects the height and top morphology, with the growth time controlling the overall height. Ag/AgCl facilitate Zn^2+^ release for sterilization without light exposure, resulting in high antibacterial efficiency when combined with ZnO. With just 3.49 μg cm^−2^ Ag/AgCl loading, the superhydrophobic composite material exhibits an antibacterial rate against *E. coli* and *S. aureus* reaching 99.03% and 99.86%, respectively. The modification of FAS molecules with low surface energy enables the composite to achieve superhydrophobic Cassie–Baxter wettability due to an air layer on its surface that reduces adhesion force—resulting in a superior bacterial repulsion effect—with anti-bacterial adhesion rates reaching 99.50% for *E. coli* and 99.70% for *S. aureus.* Therefore, this controllable method for preparing ZnO nanorod arrays with a hexagonal wurtzite structure on titanium-based surfaces effectively addresses the issues of low bactericidal efficiency without lighting, high bactericidal efficiency limited by ultraviolet photocatalysis, susceptibility to ZnO cluster formation, and easy adhesion of bacteria to its surface This breakthrough is poised to be seamlessly integrated onto diverse material surfaces, heralding a new frontier in the realm of antibacterial and anti-adhesive material research.

## Figures and Tables

**Table 1:** The specific components of as-prepared samples.

Ti@ZnO@Ag/AgCl@FAS Composite Materials		
Sample	Ag/AgCl Content	FAS Content
Ti@ZnO@Ag/AgCl@FAS(1)	3.49 ug	1 mg cm^−2^
Ti@ZnO@Ag/AgCl@FAS(2)	3.49 ug	2 mg cm^−2^
Ti@ZnO@Ag/AgCl@FAS(3)	3.49 ug	3 mg cm^−2^
Ti@ZnO@Ag/AgCl@FAS(4)	3.49 ug	4 mg cm^−2^
Ti@ZnO@Ag/AgCl@FAS(5)	3.49 ug	5 mg cm^−2^

## Data Availability

Data will be made available on request.

## Supplementary Materials

The following supporting information can be downloaded at: https://www.mdpi.com/article/10.3390/ma18071645/s1, Figure S1: (a) Top-view SEM images of Ti@ZnO with different precursor concentrations; (b) Cross-sectional SEM images of Ti@ZnO with different growth temperature; (c) Top-view SEM images of Ti@ZnO with different growth temperature; (d) Cross-sectional SEM images of Ti@ZnO with different growth time; Figure S2: Effect of precursor concentration, growth temperature and growth time on the water contact angle of Ti@ZnO@FAS surface; Figure S3: (a) SEM-EDX mapping image of Ti@ZnO@Ag/AgCl_(1)_@FAS; (b) SEM-EDX mapping image of Ti@ZnO@Ag/AgCl_(5)_@FAS; (c) SEM-EDX mapping image of Ti@ZnO@Ag/AgCl_(10)_@FAS; Figure S4: (a) Plate colony count comparison of bacterial suspension after antimicrobial testing of control, and Ti@ZnO@Ag/AgCl samples (Control represents Ti, S1 represents Ti@ZnO, S2 represents Ti@ZnO@Ag/AgCl_(1)_, S3 represents Ti@ZnO@Ag/AgCl_(5)_, S4 represents Ti@ZnO@Ag/AgCl_(10)_); (b) Plate colony count comparison of bacterial suspension after antimicrobial testing of control, and Ti@ZnO@Ag/AgCl@FAS samples (Control represents Ti, S1 represents Ti@ZnO@FAS, S2 represents Ti@ZnO@Ag/AgCl_(1)_@FAS, S3 represents Ti@ZnO@Ag/AgCl_(5)_@FAS, S4 represents Ti@ZnO@Ag/AgCl_(10)_@FAS); Figure S5: Results of plate colony count on the Ti@ZnO@Ag/AgCl@FAS after the bacterial anti-adhesive test; Figure S6: Change curve of the adhesion force on the Ti@ZnO@Ag/AgCl@FAS with different grafting densities of (a) 0.1, (b) 0.2, (c) 0.3, and (d), 0.5 mg⋅cm^−2^;

## References

[1] Zhen X., Stålsby Lundborg C., Sun X., Zhu N., Gu S., Dong H. (2021). Economic burden of antibiotic resistance in China: A national level estimate for inpatients. Antimicrob. Resist. Infect. Control.

[2] Duong T.H., Park J.W., Maeng S.K. (2021). Assessment of organic carbon migration and biofilm formation potential on polymeric tubes in contact with water. J. Hazard. Mater..

[3] Zhang X., Yang C., Yang K. (2023). Novel antibacterial metals as food contact materials: A review. Materials.

[4] Shao L., Xi Y., Weng Y. (2022). Recent advances in PLA-based antibacterial food packaging and its applications. Molecules.

[5] Song B.Y., Zhang E.S., Han X.F., Zhu H., Shi Y.J., Cao Z.Q. (2020). Engineering and application perspectives on designing an antimicrobial surface. ACS Appl. Mater. Interfaces.

[6] Murray C.J.L., Ikuta K.S., Sharara F., Swetschinski L., Robles Aguilar G., Gray A., Han C., Bisignano C., Rao P., Wool E. (2022). Global burden of bacterial antimicrobial resistance in 2019: A systematic analysis. Lancet.

[7] Xie M., Gao M., Yun Y., Malmsten M., Rotello V.M., Zboril R., Akhavan O., Kraskouski A., Amalraj J., Cai X. (2023). Antibacterial nanomaterials: Mechanisms, impacts on antimicrobial resistance and design principles. Angew. Chem. Int. Ed..

[8] Arciola C.R., Campoccia D., Montanaro L. (2018). Implant infections: Adhesion, biofilm formation and immune evasion. Nat. Rev. Microbiol..

[9] Li Y., Xu D., Chen C., Li X., Jia R., Zhang D., Sand W., Wang F., Gu T. (2018). Anaerobic microbiologically influenced corrosion mechanisms interpreted using bioenergetics and bioelectrochemistry: A review. J. Mater. Sci. Technol..

[10] Li Y., Ning C. (2019). Latest research progress of marine microbiological corrosion and bio-fouling, and new approaches of marine anti-corrosion and anti-fouling. Bioact. Mater..

[11] Grengg C., Mittermayr F., Ukrainczyk N., Koraimann G., Kienesberger S., Dietzel M. (2018). Advances in concrete materials for sewer systems affected by microbial induced concrete corrosion: A review. Water Res..

[12] Wang L., Du Y., Zhu Q., Song J., Ou K., Xie G., Yu Z. (2023). Regulating the alkyl chain length of quaternary ammonium salt to enhance the inkjet printing performance on cationic cotton fabric with reactive dye ink. ACS Appl. Mater. Interfaces.

[13] Peschel A., Otto M. (2013). Phenol-soluble modulins and staphylococcal infection. Nat. Rev. Microbiol..

[14] Pan G., Li F., He S., Li W., Wu Q., He J., Ruan R., Xiao Z., Zhang J., Yang H. (2022). Mussel-and barnacle cement proteins-inspired dual-bionic bioadhesive with repeatable wet-tissue adhesion, multimodal self-healing, and antibacterial capability for nonpressing hemostasis and promoted wound healing. Adv. Funct. Mater..

[15] Qian Y., Deng S., Cong Z., Zhang H., Lu Z., Shao N., Bhatti S.A., Zhou C., Cheng J., Gellman S.H. (2022). Secondary amine pendant β-peptide polymers displaying potent antibacterial activity and promising therapeutic potential in treating MRSA-induced wound infections and keratitis. J. Am. Chem. Soc..

[16] Xie W., Chen J., Cheng X., Feng H., Zhang X., Zhu Z., Dong S., Wan Q., Pei X., Wang J. (2023). Multi-mechanism antibacterial strategies enabled by synergistic activity of metal–organic framework-based nanosystem for infected tissue regeneration. Small.

[17] Han D., Liu X., Wu S. (2022). Metal organic framework-based antibacterial agents and their underlying mechanisms. Chem. Soc. Rev..

[18] Kaur H., Jakob R.P., Marzinek J.K., Green R., Imai Y., Bolla J.R., Agustoni E., Robinson C.V., Bond P.J., Lewis K. (2021). The antibiotic darobactin mimics a β-strand to inhibit outer membrane insertase. Nature.

[19] Du X., Wu L., Yan H., Jiang Z., Li S., Li W., Bai Y., Wang H., Cheng Z., Kong D. (2021). Microchannelled alkylated chitosan sponge to treat noncompressible hemorrhages and facilitate wound healing. Nat. Commun..

[20] Hancock R.E.W., Alford M.A., Haney E.F. (2021). Antibiofilm activity of host defence peptides: Complexity provides opportunities. Nat. Rev. Microbiol..

[21] Wang H., Wang M., Xu X., Gao P., Xu Z., Zhang Q., Li H., Yan A., Kao R.Y.-T., Sun H. (2021). Multi-target mode of action of silver against staphylococcus aureus endows it with capability to combat antibiotic resistance. Nat. Commun..

[22] Yan Y.C., Li G.F., Su M.M., Liang H. (2024). Scutellaria baicalensis polysaccharide-mediated green synthesis of smaller silver nanoparticles with enhanced antimicrobial and antibiofilm activity. ACS Appl. Mater. Interfaces.

[23] Dong C., Feng W., Xu W., Yu L., Xiang H., Chen Y., Zhou J. (2020). The coppery age: Copper (Cu)-involved nanotheranostics. Adv. Sci..

[24] Tu Y., Lv M., Xiu P., Huynh T., Zhang M., Castelli M., Liu Z., Huang Q., Fan C., Fang H. (2013). Destructive extraction of phospholipids from escherichia coli membranes by graphene nanosheets. Nat. Nanotech.

[25] Wang R., Shi M., Xu F., Qiu Y., Zhang P., Shen K., Zhao Q., Yu J., Zhang Y. (2020). Graphdiyne-modified TiO_2_ nanofibers with osteoinductive and enhanced photocatalytic antibacterial activities to prevent implant infection. Nat. Commun..

[26] Vitasovic T., Caniglia G., Eghtesadi N., Ceccato M., Bojesen E.D., Gosewinkel U., Neusser G., Rupp U., Walther P., Kranz C. (2024). Antibacterial action of Zn^2+^ ions driven by the In vivo formed ZnO nanoparticles. ACS Appl. Mater..

[27] Sirelkhatim A., Mahmud S., Seeni A., Kaus N.H.M., Ann L.C., Bakhori S.K.M., Hasan H., Mohamad D. (2015). Review on zinc oxide nanoparticles: Antibacterial activity and toxicity mechanism. Nano-Micro Lett..

[28] Brindha B., Mohammad K., Okla Kokilavani S., Sabariselvan L., Saud S.A., Mostafa A., Abdel M., Sudheer K.S. (2024). Dynamic Ag-mediated electron transfer confined ZnO nanorods for boosted photocatalytic bacterial disinfection. J. Clean. Prod.

[29] Sellappan L.K., Manoharan S. (2024). Fabrication of bioinspired keratin/sodium alginate based biopolymeric mat loaded with herbal drug and green synthesized zinc oxide nanoparticles as a dual drug antimicrobial wound dressing. Int. J. Biol. Macromol..

[30] Shrestha P., Jha M.K., Ghimire J., Koirala A.R., Shrestha R.M., Sharma R.K., Pant B., Park M., Pant H.R. (2020). Decoration of Zinc Oxide Nanorods into the Surface of Activated Carbon Obtained from Agricultural Waste for Effective Removal of Methylene Blue Dye. Materials.

[31] Prasert A., Sontikaew S., Sriprapai D., Chuangchote S. (2020). Polypropylene/ZnO Nanocomposites: Mechanical Properties, Photocatalytic Dye Degradation, and Antibacterial Property. Materials..

[32] Ma Q., Zhang H., Zhang X., Li B., Guo R., Cheng Q., Cheng X. (2019). Synthesis of magnetic CuO/MnFe_2_O_4_ nanocompisite and its high activity for degradation of levofloxacin by activation of persulfate. Chem. Eng. J..

[33] Tran T.V., Nguyen D.T.C., Kumar P.S., Din A.T.M., Jalil A.A. (2022). Vo D-VN Green synthesis of ZrO_2_ nanoparticles and nanocomposites for biomedical and environmental applications: A review. Environ. Chem. Lett..

[34] Dong S., Cui L., Tian Y., Xia L., Wu Y., Yu J., Bagley D.M., Sun J., Fan M. (2020). A novel and high-performance double Z-scheme photocatalyst ZnO-SnO_2_-Zn_2_SnO_4_ for effective removal of the biological toxicity of antibiotics. J. Hazard. Mater..

[35] Cheng C.H., Liu H.C., Lin J.C. (2021). Surface modification of polyurethane membrane with various hydrophilic monomers and N-halamine: Surface characterization and antimicrobial properties evaluation. Polymers.

[36] Chen M., Hu Q., Wang X., Zhang W. (2024). A review on recent trends of the antibacterial nonwovens air filter materials: Classification, fabrication, and application. Sep. Purif. Technol..

[37] Rabiee N., Ahmadi S., Akhavan O., Luque R. (2022). Silver and gold nanoparticles for antimicrobial purposes against multi-drug resistance bacteria. Materials.

[38] Ebrahimi M., Asadi M., Akhavan O. (2021). Graphene-based nanomaterials in fighting the most challenging viruses and immunogenic disorders. ACS Biomater. Sci. Eng..

[39] Pan Y., Zheng H., Li G., Li Y., Jiang J., Chen J., Xie Q., Wu D., Ma R., Liu X. (2022). Antibiotic-like activity of atomic layer boron nitride for combating resistant bacteria. ACS Nano.

[40] Rojas K., Canales D., Amigo N., Montoille L., Cament A., Rivas L.M., Gil-Castell O., Reyes P., Ulloa M.T., Ribes-Greus A. (2019). Effective antimicrobial materials based on low-density polyethylene (LDPE) with zinc oxide (ZnO) nanoparticles. Compos Part B Eng..

[41] Izzi M., Sportelli M.C., Torsi L., Picca R.A., Cioffi N. (2023). Synthesis and antimicrobial applications of ZnO nanostructures: A review. ACS Appl. Nano Mater..

[42] Sawai J., Igarashi H., Hashimoto A., Kokugan T., Shimizu M. (1995). Evaluation of growth inhibitory effect of ceramics powder slurry on bacteria by conductance method. J. Chem. Eng. Jpn..

[43] La D.D., Nguyen-Tri P., Le K.H., Nguyen P.T.M., Nguyen M.D.-B., Vo A.T.K., Nguyen M.T.H., Chang S.W., Tran L.D., Chung W.J. (2021). Effects of antibacterial ZnO nanoparticles on the performance of a chitosan/gum arabic edible coating for post-harvest banana preservation. Prog. Org. Coat..

[44] Leung Y.H., Xu X., Ma A.P.Y., Liu F., Ng A.M.C., Shen Z., Gethings L.A., Guo M.Y., Djurišić A.B., Lee P.K.H. (2016). Toxicity of ZnO and TiO_2_ to Escherichia coli cells. Sci. Rep..

[45] Joe A., Park S.H., Kim D.J., Lee Y.J., Jhee K.H., Sohn Y., Jang E.S. (2018). Antimicrobial activity of ZnO nanoplates and its Ag nanocomposites: Insight into an ROS-mediated antibacterial mechanism under UV light. J. Solid State Chem..

[46] Chen J., Shan M., Zhu H., Zhang S., Li J., Li L. (2023). Antimicrobial properties of heterojunction BiSnSbO_6_-ZnO composites in wastewater treatment. Environ. Sci. Pollut. Res..

[47] Raghupathi K.R., Koodali R.T., Manna A.C. (2011). Size-dependent bacterial growth inhibition and mechanism of antibacterial activity of zinc oxide nanoparticles. Langmuir.

[48] Shu Z., Zhang Y., Yang Q., Yang H. (2017). Halloysite nanotubes supported Ag and ZnO nanoparticles with synergistically enhanced antibacterial activity. Nanoscale Res. Lett..

[49] Qu B., Xiao Z., Luo Y., Luo Y. (2023). Carboxymethyl cellulose capped zinc oxide nanoparticles dispersed in ionic liquid and its antimicrobial effects against foodborne pathogens. Carbohydr. Polym. Technol. Appl..

[50] Lu H., Lin J., Lin J., Hua Z., Hu F., Ouyang L. (2023). Active bacterial anti-adhesion strategy based on directional transportation of droplet self-actuated by Laplace pressure gradient on self-actuated and infrared sensing responsive platform. Chem. Eng. J..

[51] Wang T., Huang L., Liu Y., Li X., Liu C., Handschuh-Wang S., Xu Y., Zhao Y., Tang Y. (2020). Robust biomimetic hierarchical diamond architecture with a self-cleaning, antibacterial, and antibiofouling surface. ACS Appl. Mater. Interfaces.

[52] Oh J.K., Lu X., Min Y., Cisneros-Zevallos L., Akbulut M. (2015). Bacterially Antiadhesive, Optically transparent surfaces inspired from rice leaves. ACS Appl. Mater. Interfaces.

[53] Gui L., Lin J., Liu J., Zuo J., Wang Q., Jiang W., Feng T., Li S., Wang S., Liu Z. (2022). Difference and association of antibacterial and bacterial anti-adhesive performances between smart Ag/AgCl/TiO_2_ composite surfaces with switchable wettability. Chem. Eng. J..

[54] Lin J., Cai X., Liu Z., Liu N., Xie M., Zhou B., Wang H., Guo Z. (2020). Anti-liquid-Interfering and bacterially antiadhesive strategy for highly stretchable and ultrasensitive strain sensors based on Cassie-Baxter wetting state. Adv. Funct. Mater..

[55] Qin L., Mawignon F.J., Hussain M., Ange N.K., Lu S., Hafezi M., Dong G. (2021). Economic friendly ZnO-based UV sensors using hydrothermal growth: A review. Materials.

[56] Fraleoni-Morgera A., Cesini I., Kumar P., Oddo C.M. (2019). Hydrothermally grown ZnO nanorods as promising materials for low cost electronic skin. ChemNanoMat.

[57] Xin Z., He Q., Wang S., Han X., Fu Z., Xu X., Zhao X. (2022). Recent progress in ZnO-based nanostructures for photocatalytic antimicrobial in water treatment: A review. Appl. Sci..

[58] Qamar M.T., Aslam M., Ismail I.M.I., Salah N., Hameed A. (2016). The assessment of the photocatalytic activity of magnetically retrievable ZnO coated γ-Fe_2_O_3_ in sunlight exposure. Chem. Eng. J..

[59] Abebe B., Zereffa E.A., Tadesse A., Murthy H.C.A. (2020). A review on enhancing the antibacterial activity of ZnO: Mechanisms and microscopic investigation. Nanoscale Res. Lett..

[60] Jakimińska A., Macyk W. (2023). Photochemical transformations of AgCl in the context of its eventual photocatalytic applications. J. Photochem. Photobiol. A Chem..

[61] Znaidi L., Soler Illia G.J.A.A., Benyahia S., Sanchez C., Kanaev A.V. (2003). Oriented ZnO thin films synthesis by sol–gel process for laser application. Thin Solid Films.

[62] Znaidi L. (2010). Sol–gel-deposited ZnO thin films: A review. Mater. Sci. Eng. B.

[63] Sugunan A., Warad H.C., Boman M., Dutta J. (2006). Zinc oxide nanowires in chemical bath on seeded substrates: Role of hexamine. J. Sol-Gel Sci. Technol..

[64] Amin G., Asif M.H., Zainelabdin A., Zaman S., Nur O. (2011). Willander M, Influence of pH, precursor concentration, growth time, and temperature on the morphology of ZnO nanostructures grown by the hydrothermal method. J. Nanomater..

[65] Guo M., Diao P., Wang X., Cai S. (2005). The effect of hydrothermal growth temperature on preparation and photoelectrochemical performance of ZnO nanorod array films. J. Solid State Chem..

[66] Demes T., Ternon C., Morisot F., Riassetto D., Legallais M., Roussel H., Langlet M. (2017). Mechanisms involved in the hydrothermal growth of ultra-thin and high aspect ratio ZnO nanowires. Appl. Surf. Sci..

[67] Li Q., Bian J., Sun J., Wang J., Luo Y., Sun K., Yu D. (2010). Controllable growth of well-aligned ZnO nanorod arrays by low-temperature wet chemical bath deposition method. Appl. Surf. Sci..

[68] Tian J.H., Hu J., Li S.S., Zhang F., Liu J., Shi J., Li X., Tian Z.-Q., Chen Y. (2011). Improved seedless hydrothermal synthesis of dense and ultralong ZnO nanowires. Nanotechnology.

[69] Xie Y., Yang S., Mao Z., Li P., Zhao C., Cohick Z., Huang P.-H., Huang T.J. (2014). In situ fabrication of 3D Ag@ZnO nanostructures for microfluidic surface-enhanced raman scattering systems. ACS Nano.

[70] Zhang C. (2010). High-quality oriented ZnO films grown by sol–gel process assisted with ZnO seed layer. J. Phys. Chem. Solids.

[71] Poortinga A.T., Bos R., Norde W., Busscher H.J. (2002). Electric double layer interactions in bacterial adhesion to surfaces. Surf. Sci. Rep..

[72] Wang X., Pan L., Zheng A., Cao L., Wen J., Su T., Zhang X., Huang Q., Jiang X. (2023). Multifunctionalized carbon-fiber-reinforced polyetheretherketone implant for rapid osseointegration under infected environment. Bioact. Mater..

[73] Zhang L., Bai H., Liu L., Sun D.D. (2018). Dimension induced intrinsic physio-electrical effects of nanostructured TiO_2_ on its antibacterial properties. Chem. Eng. J..

[74] Wang Y.-W., Cao A., Jiang Y., Zhang X., Liu J.-H., Liu Y., Wang H. (2014). Superior antibacterial activity of zinc oxide/graphene oxide composites originating from high zinc concentration localized around bacteria. ACS Appl. Mater. Interfaces.

[75] Li S., Dong S., Xu W., Tu S., Yan L., Zhao C., Ding J., Chen X. (2018). Antibacterial hydrogels. Adv. Sci..

[76] Moritz M., Geszke-Moritz M. (2013). The newest achievements in synthesis, immobilization and practical applications of antibacterial nanoparticles. Chem. Eng. J..

