# Supplementary material for: Controllable Fabrication of ZnO Nanorod Arrays on the Surface of Titanium Material and Their Antibacterial and Anti-Adhesion Properties
Source: Materials (Basel). 2025 Apr 3;18(7):1645. doi: 10.3390/ma18071645 (PMC11990534; doi:10.3390/ma18071645)
Supplement: Supplementary file 1 [file materials-18-01645-s001.zip › materials-3525655-supplementary.pdf]

Supplementary Information

Controllable fabrication of ZnO nanorod arrays on the surface of titanium material and their antibacterial and anti-adhesion properties

Sifang Kong,[a] Jialin Li,[b] Ouyang Fan,*[a] Feng Lin,[b] Jiayin Xie,[b] and Jing Lin,*[b]

a. School of Traffic & Environment, Shenzhen Institute of Information Technology, Shenzhen 518172, P.R. China;

b. School of Chemistry and Chemical Engineering, Guangzhou University, Guangzhou, 510006, P.R. China;

∗ Corresponding authors.

E-mail addresses: ouyangfanily@163.com (O. Fan), and linjing@gzhu.edu.cn(J. Lin).

This file includes:

Fig. S1-6

Supplementary Figures

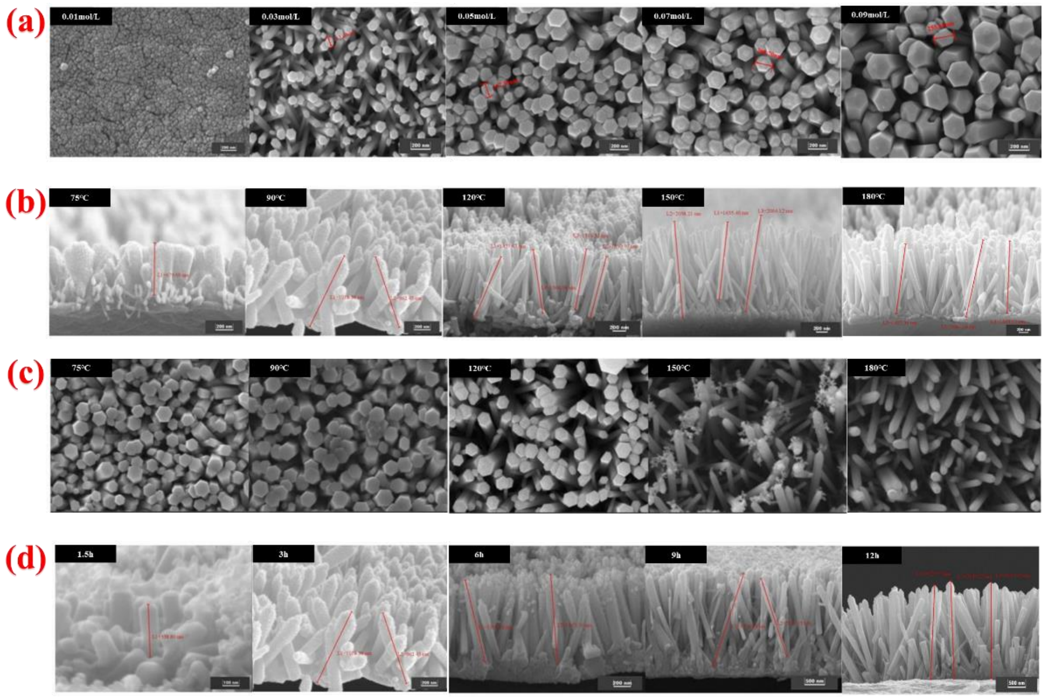

Fig. S1. a) Top-view SEM images of Ti@ZnO with different precursor concentrations; b) Cross-sectional SEM images of Ti@ZnO with different growth temperature; c) Top-view SEM images of Ti@ZnO with different growth temperature; d) Cross-sectional SEM images of Ti@ZnO with different growth time.

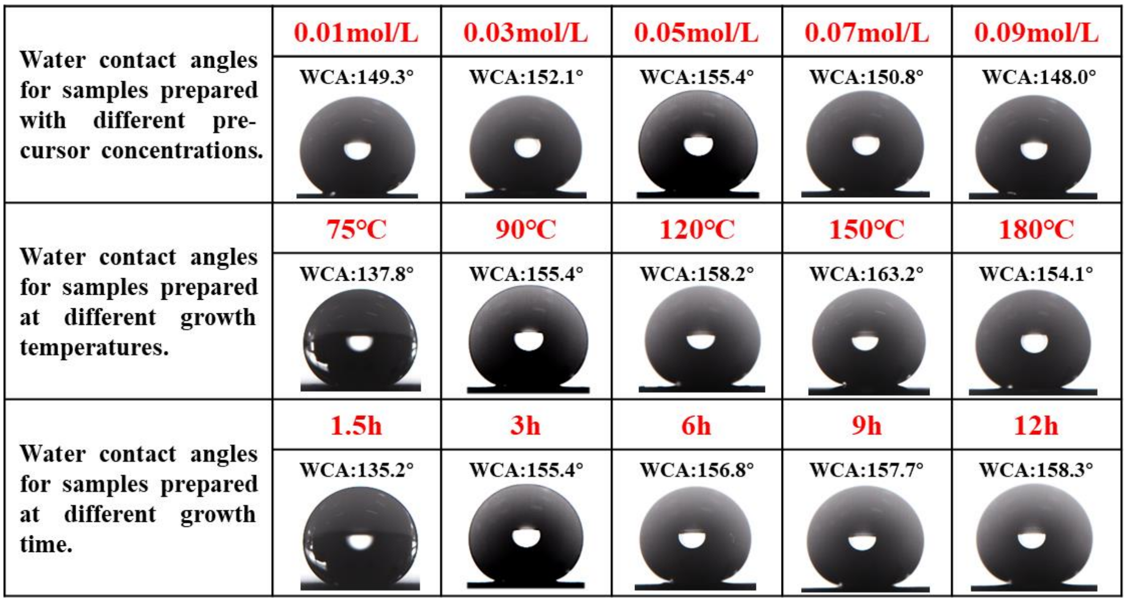

Fig. S2 Effect of precursor concentration, growth temperature and growth time on the water contact angle of Ti@ZnO@FAS surface.

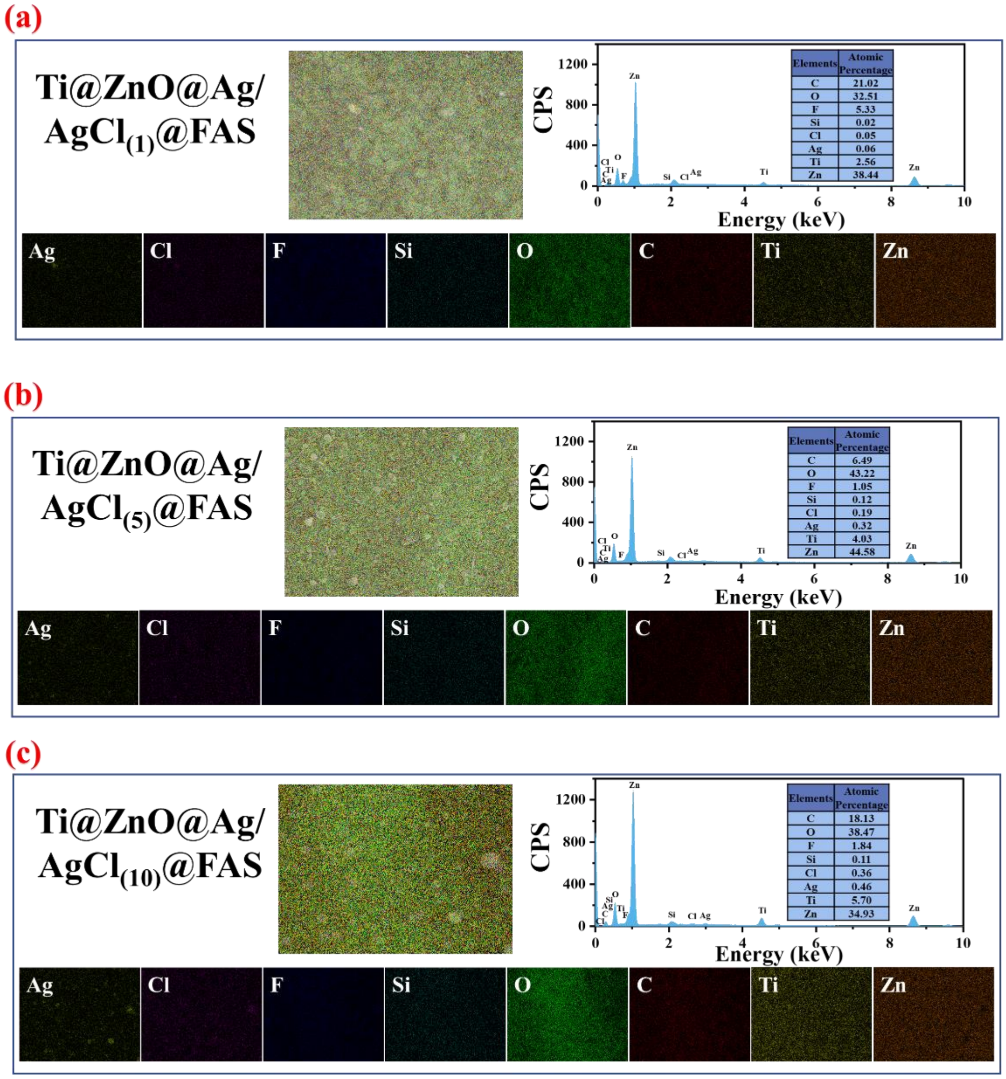

Fig. S3 a) SEM-EDX mapping image of Ti@ZnO@Ag/AgCl$_{(1)}$@FAS; b) SEM-EDX mapping image of Ti@ZnO@Ag/AgCl$_{(5)}$@FAS; c) SEM-EDX mapping image of Ti@ZnO@Ag/AgCl$_{(10)}$@FAS.

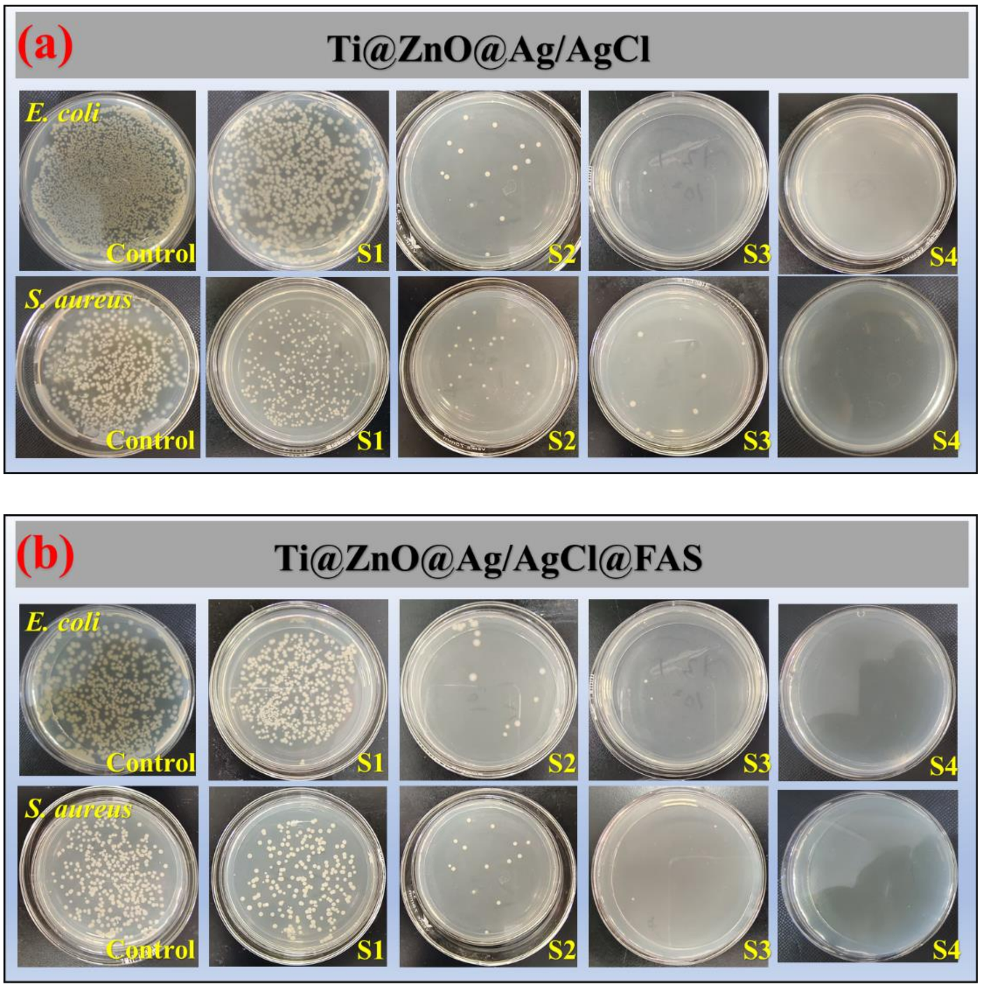

Fig. S4 a) Plate colony count comparison of bacterial suspension after antimicrobial testing of control, and Ti@ZnO@Ag/AgCl samples (Control represents Ti, S1 represents Ti@ZnO, S2 represents Ti@ZnO@Ag/AgCl$_{(1)}$, S3 represents Ti@ZnO@Ag/AgCl$_{(5)}$, S4 represents Ti@ZnO@Ag/AgCl$_{(10)}$); b) Plate colony count comparison of bacterial suspension after antimicrobial testing of control, and Ti@ZnO@Ag/AgCl@FAS samples (Control represents Ti, S1 represents Ti@ZnO@FAS, S2 represents Ti@ZnO@Ag/AgCl$_{(1)}$@FAS, S3 represents Ti@ZnO@Ag/AgCl$_{(5)}$@FAS, S4 represents Ti@ZnO@Ag/AgCl$_{(10)}$@FAS).

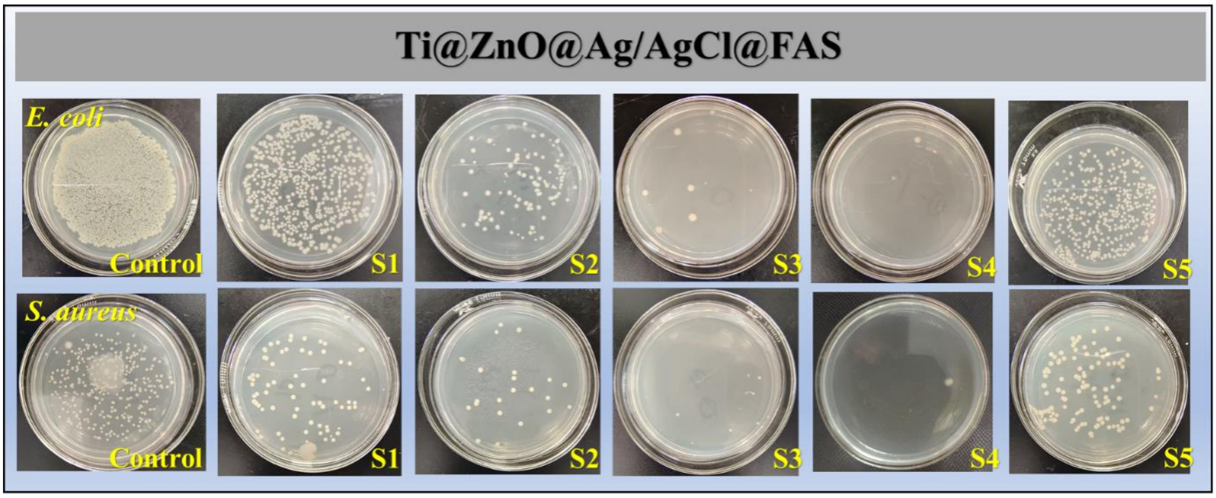

Fig. S5 Results of plate colony count on the Ti@ZnO@Ag/AgCl@FAS after the bacterial anti-adhesive test.

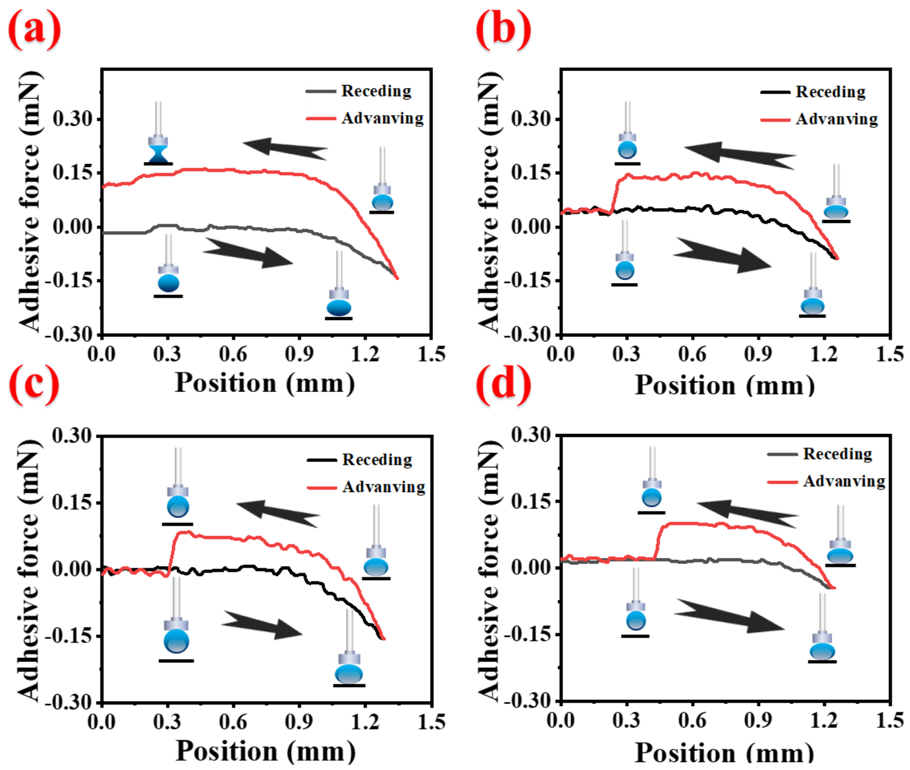

Fig. S6 Change curve of the adhesion force on the Ti@ZnO@Ag/AgCl@FAS with different grafting densities of a) 0.1, b) 0.2, c) 0.3, and d), 0.5 mg·cm^{-2}.

References

1. L. Gui, J. Lin, J. Liu, J. Zuo, Q. Wang, W. Jiang, T. Feng, S. Li, S. Wang and Z. Liu, *Chem. Eng. J.*, 2022, **431**, 134103.
